# Search for a new resonance decaying to a *W* or *Z* boson and a Higgs boson in the $\ell \ell / \ell \nu / \nu \nu + b \bar{b}$ final states with the ATLAS detector

**DOI:** 10.1140/epjc/s10052-015-3474-x

**Published:** 2015-06-16

**Authors:** G. Aad, B. Abbott, J. Abdallah, O. Abdinov, R. Aben, M. Abolins, O. S. AbouZeid, H. Abramowicz, H. Abreu, R. Abreu, Y. Abulaiti, B. S. Acharya, L. Adamczyk, D. L. Adams, J. Adelman, S. Adomeit, T. Adye, A. A. Affolder, T. Agatonovic-Jovin, J. A. Aguilar-Saavedra, S. P. Ahlen, F. Ahmadov, G. Aielli, H. Akerstedt, T. P. A. Åkesson, G. Akimoto, A. V. Akimov, G. L. Alberghi, J. Albert, S. Albrand, M. J. Alconada Verzini, M. Aleksa, I. N. Aleksandrov, C. Alexa, G. Alexander, T. Alexopoulos, M. Alhroob, G. Alimonti, L. Alio, J. Alison, S. P. Alkire, B. M. M. Allbrooke, P. P. Allport, A. Aloisio, A. Alonso, F. Alonso, C. Alpigiani, A. Altheimer, B. Alvarez Gonzalez, D. Álvarez Piqueras, M. G. Alviggi, B. T. Amadio, K. Amako, Y. Amaral Coutinho, C. Amelung, D. Amidei, S. P. Amor Dos Santos, A. Amorim, S. Amoroso, N. Amram, G. Amundsen, C. Anastopoulos, L. S. Ancu, N. Andari, T. Andeen, C. F. Anders, G. Anders, J. K. Anders, K. J. Anderson, A. Andreazza, V. Andrei, S. Angelidakis, I. Angelozzi, P. Anger, A. Angerami, F. Anghinolfi, A. V. Anisenkov, N. Anjos, A. Annovi, M. Antonelli, A. Antonov, J. Antos, F. Anulli, M. Aoki, L. Aperio Bella, G. Arabidze, Y. Arai, J. P. Araque, A. T. H. Arce, F. A Arduh, J.-F. Arguin, S. Argyropoulos, M. Arik, A. J. Armbruster, O. Arnaez, V. Arnal, H. Arnold, M. Arratia, O. Arslan, A. Artamonov, G. Artoni, S. Asai, N. Asbah, A. Ashkenazi, B. Åsman, L. Asquith, K. Assamagan, R. Astalos, M. Atkinson, N. B. Atlay, B. Auerbach, K. Augsten, M. Aurousseau, G. Avolio, B. Axen, M. K. Ayoub, G. Azuelos, M. A. Baak, A. E. Baas, C. Bacci, H. Bachacou, K. Bachas, M. Backes, M. Backhaus, E. Badescu, P. Bagiacchi, P. Bagnaia, Y. Bai, T. Bain, J. T. Baines, O. K. Baker, P. Balek, T. Balestri, F. Balli, E. Banas, Sw. Banerjee, A. A. E. Bannoura, H. S. Bansil, L. Barak, S. P. Baranov, E. L. Barberio, D. Barberis, M. Barbero, T. Barillari, M. Barisonzi, T. Barklow, N. Barlow, S. L. Barnes, B. M. Barnett, R. M. Barnett, Z. Barnovska, A. Baroncelli, G. Barone, A. J. Barr, F. Barreiro, J. Barreiro Guimarães da Costa, R. Bartoldus, A. E. Barton, P. Bartos, A. Bassalat, A. Basye, R. L. Bates, S. J. Batista, J. R. Batley, M. Battaglia, M. Bauce, F. Bauer, H. S. Bawa, J. B. Beacham, M. D. Beattie, T. Beau, P. H. Beauchemin, R. Beccherle, P. Bechtle, H. P. Beck, K. Becker, M. Becker, S. Becker, M. Beckingham, C. Becot, A. J. Beddall, A. Beddall, V. A. Bednyakov, C. P. Bee, L. J. Beemster, T. A. Beermann, M. Begel, J. K. Behr, C. Belanger-Champagne, P. J. Bell, W. H. Bell, G. Bella, L. Bellagamba, A. Bellerive, M. Bellomo, K. Belotskiy, O. Beltramello, O. Benary, D. Benchekroun, M. Bender, K. Bendtz, N. Benekos, Y. Benhammou, E. Benhar Noccioli, J. A. Benitez Garcia, D. P. Benjamin, J. R. Bensinger, S. Bentvelsen, L. Beresford, M. Beretta, D. Berge, E. Bergeaas Kuutmann, N. Berger, F. Berghaus, J. Beringer, C. Bernard, N. R. Bernard, C. Bernius, F. U. Bernlochner, T. Berry, P. Berta, C. Bertella, G. Bertoli, F. Bertolucci, C. Bertsche, D. Bertsche, M. I. Besana, G. J. Besjes, O. Bessidskaia Bylund, M. Bessner, N. Besson, C. Betancourt, S. Bethke, A. J. Beven, W. Bhimji, R. M. Bianchi, L. Bianchini, M. Bianco, O. Biebel, S. P. Bieniek, M. Biglietti, J. Bilbao De Mendizabal, H. Bilokon, M. Bindi, S. Binet, A. Bingul, C. Bini, C. W. Black, J. E. Black, K. M. Black, D. Blackburn, R. E. Blair, J.-B. Blanchard, J.E. Blanco, T. Blazek, I. Bloch, C. Blocker, W. Blum, U. Blumenschein, G. J. Bobbink, V. S. Bobrovnikov, S. S. Bocchetta, A. Bocci, C. Bock, M. Boehler, J. A. Bogaerts, A. G. Bogdanchikov, C. Bohm, V. Boisvert, T. Bold, V. Boldea, A. S. Boldyrev, M. Bomben, M. Bona, M. Boonekamp, A. Borisov, G. Borissov, S. Borroni, J. Bortfeldt, V. Bortolotto, K. Bos, D. Boscherini, M. Bosman, J. Boudreau, J. Bouffard, E. V. Bouhova-Thacker, D. Boumediene, C. Bourdarios, N. Bousson, A. Boveia, J. Boyd, I. R. Boyko, I. Bozic, J. Bracinik, A. Brandt, G. Brandt, O. Brandt, U. Bratzler, B. Brau, J. E. Brau, H. M. Braun, S. F. Brazzale, K. Brendlinger, A. J. Brennan, L. Brenner, R. Brenner, S. Bressler, K. Bristow, T. M. Bristow, D. Britton, D. Britzger, F. M. Brochu, I. Brock, R. Brock, J. Bronner, G. Brooijmans, T. Brooks, W. K. Brooks, J. Brosamer, E. Brost, J. Brown, P. A. Bruckman de Renstrom, D. Bruncko, R. Bruneliere, A. Bruni, G. Bruni, M. Bruschi, L. Bryngemark, T. Buanes, Q. Buat, P. Buchholz, A. G. Buckley, S. I. Buda, I. A. Budagov, F. Buehrer, L. Bugge, M. K. Bugge, O. Bulekov, H. Burckhart, S. Burdin, B. Burghgrave, S. Burke, I. Burmeister, E. Busato, D. Büscher, V. Büscher, P. Bussey, C. P. Buszello, J. M. Butler, A. I. Butt, C. M. Buttar, J. M. Butterworth, P. Butti, W. Buttinger, A. Buzatu, R. Buzykaev, S. Cabrera Urbán, D. Caforio, V. M. Cairo, O. Cakir, P. Calafiura, A. Calandri, G. Calderini, P. Calfayan, L. P. Caloba, D. Calvet, S. Calvet, R. Camacho Toro, S. Camarda, P. Camarri, D. Cameron, L. M. Caminada, R. Caminal Armadans, S. Campana, M. Campanelli, A. Campoverde, V. Canale, A. Canepa, M. Cano Bret, J. Cantero, R. Cantrill, T. Cao, M. D. M. Capeans Garrido, I. Caprini, M. Caprini, M. Capua, R. Caputo, R. Cardarelli, T. Carli, G. Carlino, L. Carminati, S. Caron, E. Carquin, G. D. Carrillo-Montoya, J. R. Carter, J. Carvalho, D. Casadei, M. P. Casado, M. Casolino, E. Castaneda-Miranda, A. Castelli, V. Castillo Gimenez, N. F. Castro, P. Catastini, A. Catinaccio, J. R. Catmore, A. Cattai, J. Caudron, V. Cavaliere, D. Cavalli, M. Cavalli-Sforza, V. Cavasinni, F. Ceradini, B. C. Cerio, K. Cerny, A. S. Cerqueira, A. Cerri, L. Cerrito, F. Cerutti, M. Cerv, A. Cervelli, S. A. Cetin, A. Chafaq, D. Chakraborty, I. Chalupkova, P. Chang, B. Chapleau, J. D. Chapman, D. G. Charlton, C. C. Chau, C. A. Chavez Barajas, S. Cheatham, A. Chegwidden, S. Chekanov, S. V. Chekulaev, G. A. Chelkov, M. A. Chelstowska, C. Chen, H. Chen, K. Chen, L. Chen, S. Chen, X. Chen, Y. Chen, H. C. Cheng, Y. Cheng, A. Cheplakov, E. Cheremushkina, R. Cherkaoui El Moursli, V. Chernyatin, E. Cheu, L. Chevalier, V. Chiarella, J. T. Childers, G. Chiodini, A. S. Chisholm, R. T. Chislett, A. Chitan, M. V. Chizhov, K. Choi, S. Chouridou, B. K. B. Chow, V. Christodoulou, D. Chromek-Burckhart, M. L. Chu, J. Chudoba, A. J. Chuinard, J. J. Chwastowski, L. Chytka, G. Ciapetti, A. K. Ciftci, D. Cinca, V. Cindro, I. A. Cioara, A. Ciocio, Z. H. Citron, M. Ciubancan, A. Clark, B. L. Clark, P. J. Clark, R. N. Clarke, W. Cleland, C. Clement, Y. Coadou, M. Cobal, A. Coccaro, J. Cochran, L. Coffey, J. G. Cogan, B. Cole, S. Cole, A. P. Colijn, J. Collot, T. Colombo, G. Compostella, P. Conde Muiño, E. Coniavitis, S. H. Connell, I. A. Connelly, S. M. Consonni, V. Consorti, S. Constantinescu, C. Conta, G. Conti, F. Conventi, M. Cooke, B. D. Cooper, A. M. Cooper-Sarkar, T. Cornelissen, M. Corradi, F. Corriveau, A. Corso-Radu, A. Cortes-Gonzalez, G. Cortiana, G. Costa, M. J. Costa, D. Costanzo, D. Côté, G. Cottin, G. Cowan, B. E. Cox, K. Cranmer, G. Cree, S. Crépé-Renaudin, F. Crescioli, W. A. Cribbs, M. Crispin Ortuzar, M. Cristinziani, V. Croft, G. Crosetti, T. Cuhadar Donszelmann, J. Cummings, M. Curatolo, C. Cuthbert, H. Czirr, P. Czodrowski, S. D’Auria, M. D’Onofrio, M. J. Da Cunha Sargedas De Sousa, C. Da Via, W. Dabrowski, A. Dafinca, T. Dai, O. Dale, F. Dallaire, C. Dallapiccola, M. Dam, J. R. Dandoy, N. P. Dang, A. C. Daniells, M. Danninger, M. Dano Hoffmann, V. Dao, G. Darbo, S. Darmora, J. Dassoulas, A. Dattagupta, W. Davey, C. David, T. Davidek, E. Davies, M. Davies, P. Davison, Y. Davygora, E. Dawe, I. Dawson, R. K. Daya-Ishmukhametova, K. De, R. de Asmundis, S. De Castro, S. De Cecco, N. De Groot, P. de Jong, H. De la Torre, F. De Lorenzi, L. De Nooij, D. De Pedis, A. De Salvo, U. De Sanctis, A. De Santo, J. B. De Vivie De Regie, W. J. Dearnaley, R. Debbe, C. Debenedetti, D. V. Dedovich, I. Deigaard, J. Del Peso, T. Del Prete, D. Delgove, F. Deliot, C. M. Delitzsch, M. Deliyergiyev, A. Dell’Acqua, L. Dell’Asta, M. Dell’Orso, M. Della Pietra, D. della Volpe, M. Delmastro, P. A. Delsart, C. Deluca, D. A. DeMarco, S. Demers, M. Demichev, A. Demilly, S. P. Denisov, D. Derendarz, J. E. Derkaoui, F. Derue, P. Dervan, K. Desch, C. Deterre, P. O. Deviveiros, A. Dewhurst, S. Dhaliwal, A. Di Ciaccio, L. Di Ciaccio, A. Di Domenico, C. Di Donato, A. Di Girolamo, B. Di Girolamo, A. Di Mattia, B. Di Micco, R. Di Nardo, A. Di Simone, R. Di Sipio, D. Di Valentino, C. Diaconu, M. Diamond, F. A. Dias, M. A. Diaz, E. B. Diehl, J. Dietrich, S. Diglio, A. Dimitrievska, J. Dingfelder, F. Dittus, F. Djama, T. Djobava, J. I. Djuvsland, M. A. B. do Vale, D. Dobos, M. Dobre, C. Doglioni, T. Dohmae, J. Dolejsi, Z. Dolezal, B. A. Dolgoshein, M. Donadelli, S. Donati, P. Dondero, J. Donini, J. Dopke, A. Doria, M. T. Dova, A. T. Doyle, E. Drechsler, M. Dris, E. Dubreuil, E. Duchovni, G. Duckeck, O. A. Ducu, D. Duda, A. Dudarev, L. Duflot, L. Duguid, M. Dührssen, M. Dunford, H. Duran Yildiz, M. Düren, A. Durglishvili, D. Duschinger, M. Dyndal, C. Eckardt, K. M. Ecker, R. C. Edgar, W. Edson, N. C. Edwards, W. Ehrenfeld, T. Eifert, G. Eigen, K. Einsweiler, T. Ekelof, M. El Kacimi, M. Ellert, S. Elles, F. Ellinghaus, A. A. Elliot, N. Ellis, J. Elmsheuser, M. Elsing, D. Emeliyanov, Y. Enari, O. C. Endner, M. Endo, R. Engelmann, J. Erdmann, A. Ereditato, G. Ernis, J. Ernst, M. Ernst, S. Errede, E. Ertel, M. Escalier, H. Esch, C. Escobar, B. Esposito, A. I. Etienvre, E. Etzion, H. Evans, A. Ezhilov, L. Fabbri, G. Facini, R. M. Fakhrutdinov, S. Falciano, R. J. Falla, J. Faltova, Y. Fang, M. Fanti, A. Farbin, A. Farilla, T. Farooque, S. Farrell, S. M. Farrington, P. Farthouat, F. Fassi, P. Fassnacht, D. Fassouliotis, M. Faucci Giannelli, A. Favareto, L. Fayard, P. Federic, O. L. Fedin, W. Fedorko, S. Feigl, L. Feligioni, C. Feng, E. J. Feng, H. Feng, A. B. Fenyuk, P. Fernandez Martinez, S. Fernandez Perez, S. Ferrag, J. Ferrando, A. Ferrari, P. Ferrari, R. Ferrari, D. E. Ferreira de Lima, A. Ferrer, D. Ferrere, C. Ferretti, A. Ferretto Parodi, M. Fiascaris, F. Fiedler, A. Filipčič, M. Filipuzzi, F. Filthaut, M. Fincke-Keeler, K. D. Finelli, M. C. N. Fiolhais, L. Fiorini, A. Firan, A. Fischer, C. Fischer, J. Fischer, W. C. Fisher, E. A. Fitzgerald, M. Flechl, I. Fleck, P. Fleischmann, S. Fleischmann, G. T. Fletcher, G. Fletcher, T. Flick, A. Floderus, L. R. Flores Castillo, M. J. Flowerdew, A. Formica, A. Forti, D. Fournier, H. Fox, S. Fracchia, P. Francavilla, M. Franchini, D. Francis, L. Franconi, M. Franklin, M. Fraternali, D. Freeborn, S. T. French, F. Friedrich, D. Froidevaux, J. A. Frost, C. Fukunaga, E. Fullana Torregrosa, B. G. Fulsom, J. Fuster, C. Gabaldon, O. Gabizon, A. Gabrielli, A. Gabrielli, S. Gadatsch, S. Gadomski, G. Gagliardi, P. Gagnon, C. Galea, B. Galhardo, E. J. Gallas, B. J. Gallop, P. Gallus, G. Galster, K. K. Gan, J. Gao, Y. Gao, Y. S. Gao, F. M. Garay Walls, F. Garberson, C. García, J. E. García Navarro, M. Garcia-Sciveres, R. W. Gardner, N. Garelli, V. Garonne, C. Gatti, A. Gaudiello, G. Gaudio, B. Gaur, L. Gauthier, P. Gauzzi, I. L. Gavrilenko, C. Gay, G. Gaycken, E. N. Gazis, P. Ge, Z. Gecse, C. N. P. Gee, D. A. A. Geerts, Ch. Geich-Gimbel, M. P. Geisler, C. Gemme, M. H. Genest, S. Gentile, M. George, S. George, D. Gerbaudo, A. Gershon, H. Ghazlane, B. Giacobbe, S. Giagu, V. Giangiobbe, P. Giannetti, B. Gibbard, S. M. Gibson, M. Gilchriese, T. P. S. Gillam, D. Gillberg, G. Gilles, D. M. Gingrich, N. Giokaris, M. P. Giordani, F. M. Giorgi, F. M. Giorgi, P. F. Giraud, P. Giromini, D. Giugni, C. Giuliani, M. Giulini, B. K. Gjelsten, S. Gkaitatzis, I. Gkialas, E. L. Gkougkousis, L. K. Gladilin, C. Glasman, J. Glatzer, P. C. F. Glaysher, A. Glazov, M. Goblirsch-Kolb, J. R. Goddard, J. Godlewski, S. Goldfarb, T. Golling, D. Golubkov, A. Gomes, R. Gonçalo, J. Goncalves Pinto Firmino Da Costa, L. Gonella, S. González de la Hoz, G. Gonzalez Parra, S. Gonzalez-Sevilla, L. Goossens, P. A. Gorbounov, H. A. Gordon, I. Gorelov, B. Gorini, E. Gorini, A. Gorišek, E. Gornicki, A. T. Goshaw, C. Gössling, M. I. Gostkin, D. Goujdami, A. G. Goussiou, N. Govender, H. M. X. Grabas, L. Graber, I. Grabowska-Bold, P. Grafström, K-J. Grahn, J. Gramling, E. Gramstad, S. Grancagnolo, V. Grassi, V. Gratchev, H. M. Gray, E. Graziani, Z. D. Greenwood, K. Gregersen, I. M. Gregor, P. Grenier, J. Griffiths, A. A. Grillo, K. Grimm, S. Grinstein, Ph. Gris, J.-F. Grivaz, J. P. Grohs, A. Grohsjean, E. Gross, J. Grosse-Knetter, G. C. Grossi, Z. J. Grout, L. Guan, J. Guenther, F. Guescini, D. Guest, O. Gueta, E. Guido, T. Guillemin, S. Guindon, U. Gul, C. Gumpert, J. Guo, S. Gupta, P. Gutierrez, N. G. Gutierrez Ortiz, C. Gutschow, C. Guyot, C. Gwenlan, C. B. Gwilliam, A. Haas, C. Haber, H. K. Hadavand, N. Haddad, P. Haefner, S. Hageböck, Z. Hajduk, H. Hakobyan, M. Haleem, J. Haley, D. Hall, G. Halladjian, G. D. Hallewell, K. Hamacher, P. Hamal, K. Hamano, M. Hamer, A. Hamilton, S. Hamilton, G. N. Hamity, P. G. Hamnett, L. Han, K. Hanagaki, K. Hanawa, M. Hance, P. Hanke, R. Hanna, J. B. Hansen, J. D. Hansen, M. C. Hansen, P. H. Hansen, K. Hara, A. S. Hard, T. Harenberg, F. Hariri, S. Harkusha, R. D. Harrington, P. F. Harrison, F. Hartjes, M. Hasegawa, S. Hasegawa, Y. Hasegawa, A. Hasib, S. Hassani, S. Haug, R. Hauser, L. Hauswald, M. Havranek, C. M. Hawkes, R. J. Hawkings, A. D. Hawkins, T. Hayashi, D. Hayden, C. P. Hays, J. M. Hays, H. S. Hayward, S. J. Haywood, S. J. Head, T. Heck, V. Hedberg, L. Heelan, S. Heim, T. Heim, B. Heinemann, L. Heinrich, J. Hejbal, L. Helary, S. Hellman, D. Hellmich, C. Helsens, J. Henderson, R. C. W. Henderson, Y. Heng, C. Hengler, A. Henrichs, A. M. Henriques Correia, S. Henrot-Versille, G. H. Herbert, Y. Hernández Jiménez, R. Herrberg-Schubert, G. Herten, R. Hertenberger, L. Hervas, G. G. Hesketh, N. P. Hessey, J. W. Hetherly, R. Hickling, E. Higón-Rodriguez, E. Hill, J. C. Hill, K. H. Hiller, S. J. Hillier, I. Hinchliffe, E. Hines, R. R. Hinman, M. Hirose, D. Hirschbuehl, J. Hobbs, N. Hod, M. C. Hodgkinson, P. Hodgson, A. Hoecker, M. R. Hoeferkamp, F. Hoenig, M. Hohlfeld, D. Hohn, T. R. Holmes, T. M. Hong, L. Hooft van Huysduynen, W. H. Hopkins, Y. Horii, A. J. Horton, J-Y. Hostachy, S. Hou, A. Hoummada, J. Howard, J. Howarth, M. Hrabovsky, I. Hristova, J. Hrivnac, T. Hryn’ova, A. Hrynevich, C. Hsu, P. J. Hsu, S.-C. Hsu, D. Hu, Q. Hu, X. Hu, Y. Huang, Z. Hubacek, F. Hubaut, F. Huegging, T. B. Huffman, E. W. Hughes, G. Hughes, M. Huhtinen, T. A. Hülsing, N. Huseynov, J. Huston, J. Huth, G. Iacobucci, G. Iakovidis, I. Ibragimov, L. Iconomidou-Fayard, E. Ideal, Z. Idrissi, P. Iengo, O. Igonkina, T. Iizawa, Y. Ikegami, K. Ikematsu, M. Ikeno, Y. Ilchenko, D. Iliadis, N. Ilic, Y. Inamaru, T. Ince, P. Ioannou, M. Iodice, K. Iordanidou, V. Ippolito, A. Irles Quiles, C. Isaksson, M. Ishino, M. Ishitsuka, R. Ishmukhametov, C. Issever, S. Istin, J. M. Iturbe Ponce, R. Iuppa, J. Ivarsson, W. Iwanski, H. Iwasaki, J. M. Izen, V. Izzo, S. Jabbar, B. Jackson, M. Jackson, P. Jackson, M. R. Jaekel, V. Jain, K. Jakobs, S. Jakobsen, T. Jakoubek, J. Jakubek, D. O. Jamin, D. K. Jana, E. Jansen, R. W. Jansky, J. Janssen, M. Janus, G. Jarlskog, N. Javadov, T. Javůrek, L. Jeanty, J. Jejelava, G.-Y. Jeng, D. Jennens, P. Jenni, J. Jentzsch, C. Jeske, S. Jézéquel, H. Ji, J. Jia, Y. Jiang, S. Jiggins, J. Jimenez Pena, S. Jin, A. Jinaru, O. Jinnouchi, M. D. Joergensen, P. Johansson, K. A. Johns, K. Jon-And, G. Jones, R. W. L. Jones, T. J. Jones, J. Jongmanns, P. M. Jorge, K. D. Joshi, J. Jovicevic, X. Ju, C. A. Jung, P. Jussel, A. Juste Rozas, M. Kaci, A. Kaczmarska, M. Kado, H. Kagan, M. Kagan, S. J. Kahn, E. Kajomovitz, C. W. Kalderon, S. Kama, A. Kamenshchikov, N. Kanaya, M. Kaneda, S. Kaneti, V. A. Kantserov, J. Kanzaki, B. Kaplan, A. Kapliy, D. Kar, K. Karakostas, A. Karamaoun, N. Karastathis, M. J. Kareem, M. Karnevskiy, S. N. Karpov, Z. M. Karpova, K. Karthik, V. Kartvelishvili, A. N. Karyukhin, L. Kashif, R. D. Kass, A. Kastanas, Y. Kataoka, A. Katre, J. Katzy, K. Kawagoe, T. Kawamoto, G. Kawamura, S. Kazama, V. F. Kazanin, M. Y. Kazarinov, R. Keeler, R. Kehoe, J. S. Keller, J. J. Kempster, H. Keoshkerian, O. Kepka, B. P. Kerševan, S. Kersten, R. A. Keyes, F. Khalil-zada, H. Khandanyan, A. Khanov, A.G. Kharlamov, T. J. Khoo, V. Khovanskiy, E. Khramov, J. Khubua, H. Y. Kim, H. Kim, S. H. Kim, Y. Kim, N. Kimura, O. M. Kind, B. T. King, M. King, R. S. B. King, S. B. King, J. Kirk, A. E. Kiryunin, T. Kishimoto, D. Kisielewska, F. Kiss, K. Kiuchi, O. Kivernyk, E. Kladiva, M. H. Klein, M. Klein, U. Klein, K. Kleinknecht, P. Klimek, A. Klimentov, R. Klingenberg, J. A. Klinger, T. Klioutchnikova, P. F. Klok, E.-E. Kluge, P. Kluit, S. Kluth, E. Kneringer, E. B. F. G. Knoops, A. Knue, A. Kobayashi, D. Kobayashi, T. Kobayashi, M. Kobel, M. Kocian, P. Kodys, T. Koffas, E. Koffeman, L. A. Kogan, S. Kohlmann, Z. Kohout, T. Kohriki, T. Koi, H. Kolanoski, I. Koletsou, A. A. Komar, Y. Komori, T. Kondo, N. Kondrashova, K. Köneke, A. C. König, S. König, T. Kono, R. Konoplich, N. Konstantinidis, R. Kopeliansky, S. Koperny, L. Köpke, A. K. Kopp, K. Korcyl, K. Kordas, A. Korn, A. A. Korol, I. Korolkov, E. V. Korolkova, O. Kortner, S. Kortner, T. Kosek, V. V. Kostyukhin, V. M. Kotov, A. Kotwal, A. Kourkoumeli-Charalampidi, C. Kourkoumelis, V. Kouskoura, A. Koutsman, R. Kowalewski, T. Z. Kowalski, W. Kozanecki, A. S. Kozhin, V. A. Kramarenko, G. Kramberger, D. Krasnopevtsev, M. W. Krasny, A. Krasznahorkay, J. K. Kraus, A. Kravchenko, S. Kreiss, M. Kretz, J. Kretzschmar, K. Kreutzfeldt, P. Krieger, K. Krizka, K. Kroeninger, H. Kroha, J. Kroll, J. Kroseberg, J. Krstic, U. Kruchonak, H. Krüger, N. Krumnack, Z. V. Krumshteyn, A. Kruse, M. C. Kruse, M. Kruskal, T. Kubota, H. Kucuk, S. Kuday, S. Kuehn, A. Kugel, F. Kuger, A. Kuhl, T. Kuhl, V. Kukhtin, Y. Kulchitsky, S. Kuleshov, M. Kuna, T. Kunigo, A. Kupco, H. Kurashige, Y. A. Kurochkin, R. Kurumida, V. Kus, E. S. Kuwertz, M. Kuze, J. Kvita, T. Kwan, D. Kyriazopoulos, A. La Rosa, J. L. La Rosa Navarro, L. La Rotonda, C. Lacasta, F. Lacava, J. Lacey, H. Lacker, D. Lacour, V. R. Lacuesta, E. Ladygin, R. Lafaye, B. Laforge, T. Lagouri, S. Lai, L. Lambourne, S. Lammers, C. L. Lampen, W. Lampl, E. Lançon, U. Landgraf, M. P. J. Landon, V. S. Lang, J. C. Lange, A. J. Lankford, F. Lanni, K. Lantzsch, S. Laplace, C. Lapoire, J. F. Laporte, T. Lari, F. Lasagni Manghi, M. Lassnig, P. Laurelli, W. Lavrijsen, A. T. Law, P. Laycock, O. Le Dortz, E. Le Guirriec, E. Le Menedeu, M. LeBlanc, T. LeCompte, F. Ledroit-Guillon, C. A. Lee, S. C. Lee, L. Lee, G. Lefebvre, M. Lefebvre, F. Legger, C. Leggett, A. Lehan, G. Lehmann Miotto, X. Lei, W. A. Leight, A. Leisos, A. G. Leister, M. A. L. Leite, R. Leitner, D. Lellouch, B. Lemmer, K. J. C. Leney, T. Lenz, B. Lenzi, R. Leone, S. Leone, C. Leonidopoulos, S. Leontsinis, C. Leroy, C. G. Lester, M. Levchenko, J. Levêque, D. Levin, L. J. Levinson, M. Levy, A. Lewis, A. M. Leyko, M. Leyton, B. Li, H. Li, H. L. Li, L. Li, L. Li, S. Li, Y. Li, Z. Liang, H. Liao, B. Liberti, A. Liblong, P. Lichard, K. Lie, J. Liebal, W. Liebig, C. Limbach, A. Limosani, S. C. Lin, T. H. Lin, F. Linde, B. E. Lindquist, J. T. Linnemann, E. Lipeles, A. Lipniacka, M. Lisovyi, T. M. Liss, D. Lissauer, A. Lister, A. M. Litke, B. Liu, D. Liu, J. Liu, J. B. Liu, K. Liu, L. Liu, M. Liu, M. Liu, Y. Liu, M. Livan, A. Lleres, J. Llorente Merino, S. L. Lloyd, F. Lo Sterzo, E. Lobodzinska, P. Loch, W. S. Lockman, F. K. Loebinger, A. E. Loevschall-Jensen, A. Loginov, T. Lohse, K. Lohwasser, M. Lokajicek, B. A. Long, J. D. Long, R. E. Long, K. A. Looper, L. Lopes, D. Lopez Mateos, B. Lopez Paredes, I. Lopez Paz, J. Lorenz, N. Lorenzo Martinez, M. Losada, P. Loscutoff, P. J. Lösel, X. Lou, A. Lounis, J. Love, P. A. Love, N. Lu, H. J. Lubatti, C. Luci, A. Lucotte, F. Luehring, W. Lukas, L. Luminari, O. Lundberg, B. Lund-Jensen, D. Lynn, R. Lysak, E. Lytken, H. Ma, L. L. Ma, G. Maccarrone, A. Macchiolo, C. M. Macdonald, J. Machado Miguens, D. Macina, D. Madaffari, R. Madar, H. J. Maddocks, W. F. Mader, A. Madsen, S. Maeland, T. Maeno, A. Maevskiy, E. Magradze, K. Mahboubi, J. Mahlstedt, C. Maiani, C. Maidantchik, A. A. Maier, T. Maier, A. Maio, S. Majewski, Y. Makida, N. Makovec, B. Malaescu, Pa. Malecki, V. P. Maleev, F. Malek, U. Mallik, D. Malon, C. Malone, S. Maltezos, V. M. Malyshev, S. Malyukov, J. Mamuzic, G. Mancini, B. Mandelli, L. Mandelli, I. Mandić, R. Mandrysch, J. Maneira, A. Manfredini, L. Manhaes de Andrade Filho, J. Manjarres Ramos, A. Mann, P. M. Manning, A. Manousakis-Katsikakis, B. Mansoulie, R. Mantifel, M. Mantoani, L. Mapelli, L. March, G. Marchiori, M. Marcisovsky, C. P. Marino, M. Marjanovic, F. Marroquim, S. P. Marsden, Z. Marshall, L. F. Marti, S. Marti-Garcia, B. Martin, T. A. Martin, V. J. Martin, B. Martin dit Latour, M. Martinez, S. Martin-Haugh, V. S. Martoiu, A. C. Martyniuk, M. Marx, F. Marzano, A. Marzin, L. Masetti, T. Mashimo, R. Mashinistov, J. Masik, A. L. Maslennikov, I. Massa, L. Massa, N. Massol, P. Mastrandrea, A. Mastroberardino, T. Masubuchi, P. Mättig, J. Mattmann, J. Maurer, S. J. Maxfield, D. A. Maximov, R. Mazini, S. M. Mazza, L. Mazzaferro, G. Mc Goldrick, S. P. Mc Kee, A. McCarn, R. L. McCarthy, T. G. McCarthy, N. A. McCubbin, K. W. McFarlane, J. A. Mcfayden, G. Mchedlidze, S. J. McMahon, R. A. McPherson, M. Medinnis, S. Meehan, S. Mehlhase, A. Mehta, K. Meier, C. Meineck, B. Meirose, B. R. Mellado Garcia, F. Meloni, A. Mengarelli, S. Menke, E. Meoni, K. M. Mercurio, S. Mergelmeyer, P. Mermod, L. Merola, C. Meroni, F. S. Merritt, A. Messina, J. Metcalfe, A. S. Mete, C. Meyer, C. Meyer, J-P. Meyer, J. Meyer, R. P. Middleton, S. Miglioranzi, L. Mijović, G. Mikenberg, M. Mikestikova, M. Mikuž, M. Milesi, A. Milic, D. W. Miller, C. Mills, A. Milov, D. A. Milstead, A. A. Minaenko, Y. Minami, I. A. Minashvili, A. I. Mincer, B. Mindur, M. Mineev, Y. Ming, L. M. Mir, T. Mitani, J. Mitrevski, V. A. Mitsou, A. Miucci, P. S. Miyagawa, J. U. Mjörnmark, T. Moa, K. Mochizuki, S. Mohapatra, W. Mohr, S. Molander, R. Moles-Valls, K. Mönig, C. Monini, J. Monk, E. Monnier, J. Montejo Berlingen, F. Monticelli, S. Monzani, R. W. Moore, N. Morange, D. Moreno, M. Moreno Llácer, P. Morettini, M. Morgenstern, M. Morii, M. Morinaga, V. Morisbak, S. Moritz, A. K. Morley, G. Mornacchi, J. D. Morris, S. S. Mortensen, A. Morton, L. Morvaj, H. G. Moser, M. Mosidze, J. Moss, K. Motohashi, R. Mount, E. Mountricha, S. V. Mouraviev, E. J. W. Moyse, S. Muanza, R. D. Mudd, F. Mueller, J. Mueller, K. Mueller, R. S. P. Mueller, T. Mueller, D. Muenstermann, P. Mullen, Y. Munwes, J. A. Murillo Quijada, W. J. Murray, H. Musheghyan, E. Musto, A. G. Myagkov, M. Myska, O. Nackenhorst, J. Nadal, K. Nagai, R. Nagai, Y. Nagai, K. Nagano, A. Nagarkar, Y. Nagasaka, K. Nagata, M. Nagel, E. Nagy, A. M. Nairz, Y. Nakahama, K. Nakamura, T. Nakamura, I. Nakano, H. Namasivayam, R. F. Naranjo Garcia, R. Narayan, T. Naumann, G. Navarro, R. Nayyar, H. A. Neal, P. Yu. Nechaeva, T. J. Neep, P. D. Nef, A. Negri, M. Negrini, S. Nektarijevic, C. Nellist, A. Nelson, S. Nemecek, P. Nemethy, A. A. Nepomuceno, M. Nessi, M. S. Neubauer, M. Neumann, R. M. Neves, P. Nevski, P. R. Newman, D. H. Nguyen, R. B. Nickerson, R. Nicolaidou, B. Nicquevert, J. Nielsen, N. Nikiforou, A. Nikiforov, V. Nikolaenko, I. Nikolic-Audit, K. Nikolopoulos, J. K. Nilsen, P. Nilsson, Y. Ninomiya, A. Nisati, R. Nisius, T. Nobe, M. Nomachi, I. Nomidis, T. Nooney, S. Norberg, M. Nordberg, O. Novgorodova, S. Nowak, M. Nozaki, L. Nozka, K. Ntekas, G. Nunes Hanninger, T. Nunnemann, E. Nurse, F. Nuti, B. J. O’Brien, F. O’grady, D. C. O’Neil, V. O’Shea, F. G. Oakham, H. Oberlack, T. Obermann, J. Ocariz, A. Ochi, I. Ochoa, S. Oda, S. Odaka, H. Ogren, A. Oh, S. H. Oh, C. C. Ohm, H. Ohman, H. Oide, W. Okamura, H. Okawa, Y. Okumura, T. Okuyama, A. Olariu, S. A. Olivares Pino, D. Oliveira Damazio, E. Oliver Garcia, A. Olszewski, J. Olszowska, A. Onofre, P. U. E. Onyisi, C. J. Oram, M. J. Oreglia, Y. Oren, D. Orestano, N. Orlando, C. Oropeza Barrera, R. S. Orr, B. Osculati, R. Ospanov, G. Otero y Garzon, H. Otono, M. Ouchrif, E. A. Ouellette, F. Ould-Saada, A. Ouraou, K. P. Oussoren, Q. Ouyang, A. Ovcharova, M. Owen, R. E. Owen, V. E. Ozcan, N. Ozturk, K. Pachal, A. Pacheco Pages, C. Padilla Aranda, M. Pagáčová, S. Pagan Griso, E. Paganis, C. Pahl, F. Paige, P. Pais, K. Pajchel, G. Palacino, S. Palestini, M. Palka, D. Pallin, A. Palma, Y. B. Pan, E. Panagiotopoulou, C. E. Pandini, J. G. Panduro Vazquez, P. Pani, S. Panitkin, L. Paolozzi, Th. D. Papadopoulou, K. Papageorgiou, A. Paramonov, D. Paredes Hernandez, M. A. Parker, K. A. Parker, F. Parodi, J. A. Parsons, U. Parzefall, E. Pasqualucci, S. Passaggio, F. Pastore, Fr. Pastore, G. Pásztor, S. Pataraia, N. D. Patel, J. R. Pater, T. Pauly, J. Pearce, B. Pearson, L. E. Pedersen, M. Pedersen, S. Pedraza Lopez, R. Pedro, S. V. Peleganchuk, D. Pelikan, H. Peng, B. Penning, J. Penwell, D. V. Perepelitsa, E. Perez Codina, M. T. Pérez García-Estañ, L. Perini, H. Pernegger, S. Perrella, R. Peschke, V. D. Peshekhonov, K. Peters, R. F. Y. Peters, B. A. Petersen, T. C. Petersen, E. Petit, A. Petridis, C. Petridou, E. Petrolo, F. Petrucci, N. E. Pettersson, R. Pezoa, P. W. Phillips, G. Piacquadio, E. Pianori, A. Picazio, E. Piccaro, M. Piccinini, M. A. Pickering, R. Piegaia, D. T. Pignotti, J. E. Pilcher, A. D. Pilkington, J. Pina, M. Pinamonti, J. L. Pinfold, A. Pingel, B. Pinto, S. Pires, M. Pitt, C. Pizio, L. Plazak, M.-A. Pleier, V. Pleskot, E. Plotnikova, P. Plucinski, D. Pluth, R. Poettgen, L. Poggioli, D. Pohl, G. Polesello, A. Policicchio, R. Polifka, A. Polini, C. S. Pollard, V. Polychronakos, K. Pommès, L. Pontecorvo, B. G. Pope, G. A. Popeneciu, D. S. Popovic, A. Poppleton, S. Pospisil, K. Potamianos, I. N. Potrap, C. J. Potter, C. T. Potter, G. Poulard, J. Poveda, V. Pozdnyakov, P. Pralavorio, A. Pranko, S. Prasad, S. Prell, D. Price, L. E. Price, M. Primavera, S. Prince, M. Proissl, K. Prokofiev, F. Prokoshin, E. Protopapadaki, S. Protopopescu, J. Proudfoot, M. Przybycien, E. Ptacek, D. Puddu, E. Pueschel, D. Puldon, M. Purohit, P. Puzo, J. Qian, G. Qin, Y. Qin, A. Quadt, D. R. Quarrie, W. B. Quayle, M. Queitsch-Maitland, D. Quilty, S. Raddum, V. Radeka, V. Radescu, S. K. Radhakrishnan, P. Radloff, P. Rados, F. Ragusa, G. Rahal, S. Rajagopalan, M. Rammensee, C. Rangel-Smith, F. Rauscher, S. Rave, T. Ravenscroft, M. Raymond, A. L. Read, N. P. Readioff, D. M. Rebuzzi, A. Redelbach, G. Redlinger, R. Reece, K. Reeves, L. Rehnisch, H. Reisin, M. Relich, C. Rembser, H. Ren, A. Renaud, M. Rescigno, S. Resconi, O. L. Rezanova, P. Reznicek, R. Rezvani, R. Richter, S. Richter, E. Richter-Was, O. Ricken, M. Ridel, P. Rieck, C. J. Riegel, J. Rieger, M. Rijssenbeek, A. Rimoldi, L. Rinaldi, B. Ristić, E. Ritsch, I. Riu, F. Rizatdinova, E. Rizvi, S. H. Robertson, A. Robichaud-Veronneau, D. Robinson, J. E. M. Robinson, A. Robson, C. Roda, S. Roe, O. Røhne, S. Rolli, A. Romaniouk, M. Romano, S. M. Romano Saez, E. Romero Adam, N. Rompotis, M. Ronzani, L. Roos, E. Ros, S. Rosati, K. Rosbach, P. Rose, P. L. Rosendahl, O. Rosenthal, V. Rossetti, E. Rossi, L. P. Rossi, R. Rosten, M. Rotaru, I. Roth, J. Rothberg, D. Rousseau, C. R. Royon, A. Rozanov, Y. Rozen, X. Ruan, F. Rubbo, I. Rubinskiy, V. I. Rud, C. Rudolph, M. S. Rudolph, F. Rühr, A. Ruiz-Martinez, Z. Rurikova, N. A. Rusakovich, A. Ruschke, H. L. Russell, J. P. Rutherfoord, N. Ruthmann, Y. F. Ryabov, M. Rybar, G. Rybkin, N. C. Ryder, A. F. Saavedra, G. Sabato, S. Sacerdoti, A. Saddique, H. F-W. Sadrozinski, R. Sadykov, F. Safai Tehrani, M. Saimpert, H. Sakamoto, Y. Sakurai, G. Salamanna, A. Salamon, M. Saleem, D. Salek, P. H. Sales De Bruin, D. Salihagic, A. Salnikov, J. Salt, D. Salvatore, F. Salvatore, A. Salvucci, A. Salzburger, D. Sampsonidis, A. Sanchez, J. Sánchez, V. Sanchez Martinez, H. Sandaker, R. L. Sandbach, H. G. Sander, M. P. Sanders, M. Sandhoff, C. Sandoval, R. Sandstroem, D. P. C. Sankey, M. Sannino, A. Sansoni, C. Santoni, R. Santonico, H. Santos, I. Santoyo Castillo, K. Sapp, A. Sapronov, J. G. Saraiva, B. Sarrazin, O. Sasaki, Y. Sasaki, K. Sato, G. Sauvage, E. Sauvan, G. Savage, P. Savard, C. Sawyer, L. Sawyer, J. Saxon, C. Sbarra, A. Sbrizzi, T. Scanlon, D. A. Scannicchio, M. Scarcella, V. Scarfone, J. Schaarschmidt, P. Schacht, D. Schaefer, R. Schaefer, J. Schaeffer, S. Schaepe, S. Schaetzel, U. Schäfer, A. C. Schaffer, D. Schaile, R. D. Schamberger, V. Scharf, V. A. Schegelsky, D. Scheirich, M. Schernau, C. Schiavi, C. Schillo, M. Schioppa, S. Schlenker, E. Schmidt, K. Schmieden, C. Schmitt, S. Schmitt, S. Schmitt, B. Schneider, Y. J. Schnellbach, U. Schnoor, L. Schoeffel, A. Schoening, B. D. Schoenrock, E. Schopf, A. L. S. Schorlemmer, M. Schott, D. Schouten, J. Schovancova, S. Schramm, M. Schreyer, C. Schroeder, N. Schuh, M. J. Schultens, H.-C. Schultz-Coulon, H. Schulz, M. Schumacher, B. A. Schumm, Ph. Schune, C. Schwanenberger, A. Schwartzman, T. A. Schwarz, Ph. Schwegler, Ph. Schwemling, R. Schwienhorst, J. Schwindling, T. Schwindt, M. Schwoerer, F. G. Sciacca, E. Scifo, G. Sciolla, F. Scuri, F. Scutti, J. Searcy, G. Sedov, E. Sedykh, P. Seema, S. C. Seidel, A. Seiden, F. Seifert, J. M. Seixas, G. Sekhniaidze, K. Sekhon, S. J. Sekula, K. E. Selbach, D. M. Seliverstov, N. Semprini-Cesari, C. Serfon, L. Serin, L. Serkin, T. Serre, M. Sessa, R. Seuster, H. Severini, T. Sfiligoj, F. Sforza, A. Sfyrla, E. Shabalina, M. Shamim, L. Y. Shan, R. Shang, J. T. Shank, M. Shapiro, P. B. Shatalov, K. Shaw, S. M. Shaw, A. Shcherbakova, C. Y. Shehu, P. Sherwood, L. Shi, S. Shimizu, C. O. Shimmin, M. Shimojima, M. Shiyakova, A. Shmeleva, D. Shoaleh Saadi, M. J. Shochet, S. Shojaii, S. Shrestha, E. Shulga, M. A. Shupe, S. Shushkevich, P. Sicho, O. Sidiropoulou, D. Sidorov, A. Sidoti, F. Siegert, Dj. Sijacki, J. Silva, Y. Silver, S. B. Silverstein, V. Simak, O. Simard, Lj. Simic, S. Simion, E. Simioni, B. Simmons, D. Simon, R. Simoniello, P. Sinervo, N. B. Sinev, G. Siragusa, A. N. Sisakyan, S. Yu. Sivoklokov, J. Sjölin, T. B. Sjursen, M. B. Skinner, H. P. Skottowe, P. Skubic, M. Slater, T. Slavicek, M. Slawinska, K. Sliwa, V. Smakhtin, B. H. Smart, L. Smestad, S. Yu. Smirnov, Y. Smirnov, L. N. Smirnova, O. Smirnova, M. N. K. Smith, M. Smizanska, K. Smolek, A. A. Snesarev, G. Snidero, S. Snyder, R. Sobie, F. Socher, A. Soffer, D. A. Soh, C. A. Solans, M. Solar, J. Solc, E. Yu. Soldatov, U. Soldevila, A. A. Solodkov, A. Soloshenko, O. V. Solovyanov, V. Solovyev, P. Sommer, H. Y. Song, N. Soni, A. Sood, A. Sopczak, B. Sopko, V. Sopko, V. Sorin, D. Sosa, M. Sosebee, C. L. Sotiropoulou, R. Soualah, P. Soueid, A. M. Soukharev, D. South, S. Spagnolo, M. Spalla, F. Spanò, W. R. Spearman, F. Spettel, R. Spighi, G. Spigo, L. A. Spiller, M. Spousta, T. Spreitzer, R. D. St. Denis, S. Staerz, J. Stahlman, R. Stamen, S. Stamm, E. Stanecka, C. Stanescu, M. Stanescu-Bellu, M. M. Stanitzki, S. Stapnes, E. A. Starchenko, J. Stark, P. Staroba, P. Starovoitov, R. Staszewski, P. Stavina, P. Steinberg, B. Stelzer, H. J. Stelzer, O. Stelzer-Chilton, H. Stenzel, S. Stern, G. A. Stewart, J. A. Stillings, M. C. Stockton, M. Stoebe, G. Stoicea, P. Stolte, S. Stonjek, A. R. Stradling, A. Straessner, M. E. Stramaglia, J. Strandberg, S. Strandberg, A. Strandlie, E. Strauss, M. Strauss, P. Strizenec, R. Ströhmer, D. M. Strom, R. Stroynowski, A. Strubig, S. A. Stucci, B. Stugu, N. A. Styles, D. Su, J. Su, R. Subramaniam, A. Succurro, Y. Sugaya, C. Suhr, M. Suk, V. V. Sulin, S. Sultansoy, T. Sumida, S. Sun, X. Sun, J. E. Sundermann, K. Suruliz, G. Susinno, M. R. Sutton, S. Suzuki, Y. Suzuki, M. Svatos, S. Swedish, M. Swiatlowski, I. Sykora, T. Sykora, D. Ta, C. Taccini, K. Tackmann, J. Taenzer, A. Taffard, R. Tafirout, N. Taiblum, H. Takai, R. Takashima, H. Takeda, T. Takeshita, Y. Takubo, M. Talby, A. A. Talyshev, J. Y. C. Tam, K. G. Tan, J. Tanaka, R. Tanaka, S. Tanaka, B. B. Tannenwald, N. Tannoury, S. Tapprogge, S. Tarem, F. Tarrade, G. F. Tartarelli, P. Tas, M. Tasevsky, T. Tashiro, E. Tassi, A. Tavares Delgado, Y. Tayalati, F. E. Taylor, G. N. Taylor, W. Taylor, F. A. Teischinger, M. Teixeira Dias Castanheira, P. Teixeira-Dias, K. K. Temming, H. Ten Kate, P. K. Teng, J. J. Teoh, F. Tepel, S. Terada, K. Terashi, J. Terron, S. Terzo, M. Testa, R. J. Teuscher, J. Therhaag, T. Theveneaux-Pelzer, J. P. Thomas, J. Thomas-Wilsker, E. N. Thompson, P. D. Thompson, R. J. Thompson, A. S. Thompson, L. A. Thomsen, E. Thomson, M. Thomson, R. P. Thun, M. J. Tibbetts, R. E. Ticse Torres, V. O. Tikhomirov, Yu. A. Tikhonov, S. Timoshenko, E. Tiouchichine, P. Tipton, S. Tisserant, T. Todorov, S. Todorova-Nova, J. Tojo, S. Tokár, K. Tokushuku, K. Tollefson, E. Tolley, L. Tomlinson, M. Tomoto, L. Tompkins, K. Toms, E. Torrence, H. Torres, E. Torró Pastor, J. Toth, F. Touchard, D. R. Tovey, T. Trefzger, L. Tremblet, A. Tricoli, I. M. Trigger, S. Trincaz-Duvoid, M. F. Tripiana, W. Trischuk, B. Trocmé, C. Troncon, M. Trottier-McDonald, M. Trovatelli, P. True, L. Truong, M. Trzebinski, A. Trzupek, C. Tsarouchas, J. C-L. Tseng, P. V. Tsiareshka, D. Tsionou, G. Tsipolitis, N. Tsirintanis, S. Tsiskaridze, V. Tsiskaridze, E. G. Tskhadadze, I. I. Tsukerman, V. Tsulaia, S. Tsuno, D. Tsybychev, A. Tudorache, V. Tudorache, A. N. Tuna, S. A. Tupputi, S. Turchikhin, D. Turecek, R. Turra, A. J. Turvey, P. M. Tuts, A. Tykhonov, M. Tylmad, M. Tyndel, I. Ueda, R. Ueno, M. Ughetto, M. Ugland, M. Uhlenbrock, F. Ukegawa, G. Unal, A. Undrus, G. Unel, F. C. Ungaro, Y. Unno, C. Unverdorben, J. Urban, P. Urquijo, P. Urrejola, G. Usai, A. Usanova, L. Vacavant, V. Vacek, B. Vachon, C. Valderanis, N. Valencic, S. Valentinetti, A. Valero, L. Valery, S. Valkar, E. Valladolid Gallego, S. Vallecorsa, J. A. Valls Ferrer, W. Van Den Wollenberg, P. C. Van Der Deijl, R. van der Geer, H. van der Graaf, R. Van Der Leeuw, N. van Eldik, P. van Gemmeren, J. Van Nieuwkoop, I. van Vulpen, M. C. van Woerden, M. Vanadia, W. Vandelli, R. Vanguri, A. Vaniachine, F. Vannucci, G. Vardanyan, R. Vari, E. W. Varnes, T. Varol, D. Varouchas, A. Vartapetian, K. E. Varvell, F. Vazeille, T. Vazquez Schroeder, J. Veatch, F. Veloso, T. Velz, S. Veneziano, A. Ventura, D. Ventura, M. Venturi, N. Venturi, A. Venturini, V. Vercesi, M. Verducci, W. Verkerke, J. C. Vermeulen, A. Vest, M. C. Vetterli, O. Viazlo, I. Vichou, T. Vickey, O. E. Vickey Boeriu, G. H. A. Viehhauser, S. Viel, R. Vigne, M. Villa, M. Villaplana Perez, E. Vilucchi, M. G. Vincter, V. B. Vinogradov, I. Vivarelli, F. Vives Vaque, S. Vlachos, D. Vladoiu, M. Vlasak, M. Vogel, P. Vokac, G. Volpi, M. Volpi, H. von der Schmitt, H. von Radziewski, E. von Toerne, V. Vorobel, K. Vorobev, M. Vos, R. Voss, J. H. Vossebeld, N. Vranjes, M. Vranjes Milosavljevic, V. Vrba, M. Vreeswijk, R. Vuillermet, I. Vukotic, Z. Vykydal, P. Wagner, W. Wagner, H. Wahlberg, S. Wahrmund, J. Wakabayashi, J. Walder, R. Walker, W. Walkowiak, C. Wang, F. Wang, H. Wang, H. Wang, J. Wang, J. Wang, K. Wang, R. Wang, S. M. Wang, T. Wang, X. Wang, C. Wanotayaroj, A. Warburton, C. P. Ward, D. R. Wardrope, M. Warsinsky, A. Washbrook, C. Wasicki, P. M. Watkins, A. T. Watson, I. J. Watson, M. F. Watson, G. Watts, S. Watts, B. M. Waugh, S. Webb, M. S. Weber, S. W. Weber, J. S. Webster, A. R. Weidberg, B. Weinert, J. Weingarten, C. Weiser, H. Weits, P. S. Wells, T. Wenaus, T. Wengler, S. Wenig, N. Wermes, M. Werner, P. Werner, M. Wessels, J. Wetter, K. Whalen, A. M. Wharton, A. White, M. J. White, R. White, S. White, D. Whiteson, F. J. Wickens, W. Wiedenmann, M. Wielers, P. Wienemann, C. Wiglesworth, L. A. M. Wiik-Fuchs, A. Wildauer, H. G. Wilkens, H. H. Williams, S. Williams, C. Willis, S. Willocq, A. Wilson, J. A. Wilson, I. Wingerter-Seez, F. Winklmeier, B. T. Winter, M. Wittgen, J. Wittkowski, S. J. Wollstadt, M. W. Wolter, H. Wolters, B. K. Wosiek, J. Wotschack, M. J. Woudstra, K. W. Wozniak, M. Wu, M. Wu, S. L. Wu, X. Wu, Y. Wu, T. R. Wyatt, B. M. Wynne, S. Xella, D. Xu, L. Xu, B. Yabsley, S. Yacoob, R. Yakabe, M. Yamada, Y. Yamaguchi, A. Yamamoto, S. Yamamoto, T. Yamanaka, K. Yamauchi, Y. Yamazaki, Z. Yan, H. Yang, H. Yang, Y. Yang, L. Yao, W-M. Yao, Y. Yasu, E. Yatsenko, K. H. Yau Wong, J. Ye, S. Ye, I. Yeletskikh, A. L. Yen, E. Yildirim, K. Yorita, R. Yoshida, K. Yoshihara, C. Young, C. J. S. Young, S. Youssef, D. R. Yu, J. Yu, J. M. Yu, J. Yu, L. Yuan, A. Yurkewicz, I. Yusuff, B. Zabinski, R. Zaidan, A. M. Zaitsev, J. Zalieckas, A. Zaman, S. Zambito, L. Zanello, D. Zanzi, C. Zeitnitz, M. Zeman, A. Zemla, K. Zengel, O. Zenin, T. Ženiš, D. Zerwas, D. Zhang, F. Zhang, J. Zhang, L. Zhang, R. Zhang, X. Zhang, Z. Zhang, X. Zhao, Y. Zhao, Z. Zhao, A. Zhemchugov, J. Zhong, B. Zhou, C. Zhou, L. Zhou, L. Zhou, N. Zhou, C. G. Zhu, H. Zhu, J. Zhu, Y. Zhu, X. Zhuang, K. Zhukov, A. Zibell, D. Zieminska, N. I. Zimine, C. Zimmermann, S. Zimmermann, Z. Zinonos, M. Zinser, M. Ziolkowski, L. Živković, G. Zobernig, A. Zoccoli, M. zur Nedden, G. Zurzolo, L. Zwalinski, ATLAS collaboration

**Affiliations:** Department of Physics, University of Adelaide, Adelaide, Australia; Physics Department, SUNY Albany, Albany, NY USA; Department of Physics, University of Alberta, Edmonton, AB Canada; Department of Physics, Ankara University, Ankara, Turkey; LAPP, CNRS/IN2P3 and Université Savoie Mont Blanc, Annecy-le-Vieux, France; High Energy Physics Division, Argonne National Laboratory, Argonne, IL USA; Department of Physics, University of Arizona, Tucson, AZ USA; Department of Physics, The University of Texas at Arlington, Arlington, TX USA; Physics Department, University of Athens, Athens, Greece; Physics Department, National Technical University of Athens, Zografou, Greece; Institute of Physics, Azerbaijan Academy of Sciences, Baku, Azerbaijan; Institut de Física d’Altes Energies and Departament de Física de la Universitat Autònoma de Barcelona, Barcelona, Spain; Institute of Physics, University of Belgrade, Belgrade, Serbia; Department for Physics and Technology, University of Bergen, Bergen, Norway; Physics Division, Lawrence Berkeley National Laboratory and University of California, Berkeley, CA USA; Department of Physics, Humboldt University, Berlin, Germany; Albert Einstein Center for Fundamental Physics and Laboratory for High Energy Physics, University of Bern, Bern, Switzerland; School of Physics and Astronomy, University of Birmingham, Birmingham, UK; Department of Physics, Bogazici University, Istanbul, Turkey; INFN Sezione di Bologna, Bologna, Italy; Physikalisches Institut, University of Bonn, Bonn, Germany; Department of Physics, Boston University, Boston, MA USA; Department of Physics, Brandeis University, Waltham, MA USA; Universidade Federal do Rio De Janeiro COPPE/EE/IF, Rio de Janeiro, Brazil; Physics Department, Brookhaven National Laboratory, Upton, NY USA; National Institute of Physics and Nuclear Engineering, Bucharest, Romania; Departamento de Física, Universidad de Buenos Aires, Buenos Aires, Argentina; Cavendish Laboratory, University of Cambridge, Cambridge, UK; Department of Physics, Carleton University, Ottawa, ON Canada; CERN, Geneva, Switzerland; Enrico Fermi Institute, University of Chicago, Chicago, IL USA; Departamento de Física, Pontificia Universidad Católica de Chile, Santiago, Chile; Institute of High Energy Physics, Chinese Academy of Sciences, Beijing, China; Laboratoire de Physique Corpusculaire, Clermont Université and Université Blaise Pascal and CNRS/IN2P3, Clermont-Ferrand, France; Nevis Laboratory, Columbia University, Irvington, NY USA; Niels Bohr Institute, University of Copenhagen, Copenhagen, Denmark; INFN Gruppo Collegato di Cosenza, Laboratori Nazionali di Frascati, Frascati, Italy; AGH University of Science and Technology, Faculty of Physics and Applied Computer Science, Krakow, Poland; Institute of Nuclear Physics, Polish Academy of Sciences, Kraków, Poland; Physics Department, Southern Methodist University, Dallas, TX USA; Physics Department, University of Texas at Dallas, Richardson, TX USA; DESY, Hamburg and Zeuthen, Germany; Institut für Experimentelle Physik IV, Technische Universität Dortmund, Dortmund, Germany; Institut für Kern- und Teilchenphysik, Technische Universität Dresden, Dresden, Germany; Department of Physics, Duke University, Durham, NC USA; SUPA, School of Physics and Astronomy, University of Edinburgh, Edinburgh, UK; INFN Laboratori Nazionali di Frascati, Frascati, Italy; Fakultät für Mathematik und Physik, Albert-Ludwigs-Universität, Freiburg, Germany; Section de Physique, Université de Genève, Geneva, Switzerland; INFN Sezione di Genova, Genova, Italy; E. Andronikashvili Institute of Physics, Iv. Javakhishvili Tbilisi State University, Tbilisi, Georgia; II Physikalisches Institut, Justus-Liebig-Universität Giessen, Giessen, Germany; SUPA, School of Physics and Astronomy, University of Glasgow, Glasgow, UK; II Physikalisches Institut, Georg-August-Universität, Göttingen, Germany; Laboratoire de Physique Subatomique et de Cosmologie, Université Grenoble-Alpes, CNRS/IN2P3, Grenoble, France; Department of Physics, Hampton University, Hampton, VA USA; Laboratory for Particle Physics and Cosmology, Harvard University, Cambridge, MA USA; Kirchhoff-Institut für Physik, Ruprecht-Karls-Universität Heidelberg, Heidelberg, Germany; Faculty of Applied Information Science, Hiroshima Institute of Technology, Hiroshima, Japan; Department of Physics, The Chinese University of Hong Kong, Shatin, NT China; Department of Physics, Indiana University, Bloomington, IN USA; Institut für Astro- und Teilchenphysik, Leopold-Franzens-Universität, Innsbruck, Austria; University of Iowa, Iowa City, IA USA; Department of Physics and Astronomy, Iowa State University, Ames, IA USA; Joint Institute for Nuclear Research, JINR Dubna, Dubna, Russia; KEK, High Energy Accelerator Research Organization, Tsukuba, Japan; Graduate School of Science, Kobe University, Kobe, Japan; Faculty of Science, Kyoto University, Kyoto, Japan; Kyoto University of Education, Kyoto, Japan; Department of Physics, Kyushu University, Fukuoka, Japan; Instituto de Física La Plata, Universidad Nacional de La Plata and CONICET, La Plata, Argentina; Physics Department, Lancaster University, Lancaster, UK; INFN Sezione di Lecce, Lecce, Italy; Oliver Lodge Laboratory, University of Liverpool, Liverpool, UK; Department of Physics, Jožef Stefan Institute and University of Ljubljana, Ljubljana, Slovenia; School of Physics and Astronomy, Queen Mary University of London, London, UK; Department of Physics, Royal Holloway University of London, Surrey, UK; Department of Physics and Astronomy, University College London, London, UK; Louisiana Tech University, Ruston, LA USA; Laboratoire de Physique Nucléaire et de Hautes Energies, UPMC and Université Paris-Diderot and CNRS/IN2P3, Paris, France; Fysiska institutionen, Lunds universitet, Lund, Sweden; Departamento de Fisica Teorica C-15, Universidad Autonoma de Madrid, Madrid, Spain; Institut für Physik, Universität Mainz, Mainz, Germany; School of Physics and Astronomy, University of Manchester, Manchester, UK; CPPM, Aix-Marseille Université and CNRS/IN2P3, Marseille, France; Department of Physics, University of Massachusetts, Amherst, MA USA; Department of Physics, McGill University, Montreal, QC Canada; School of Physics, University of Melbourne, Melbourne, VIC Australia; Department of Physics, The University of Michigan, Ann Arbor, MI USA; Department of Physics and Astronomy, Michigan State University, East Lansing, MI USA; NFN Sezione di Milano, Milan, Italy; B.I. Stepanov Institute of Physics, National Academy of Sciences of Belarus, Minsk, Republic of Belarus; National Scientific and Educational Centre for Particle and High Energy Physics, Minsk, Republic of Belarus; Department of Physics, Massachusetts Institute of Technology, Cambridge, MA USA; Group of Particle Physics, University of Montreal, Montreal, QC Canada; P.N. Lebedev Institute of Physics, Academy of Sciences, Moscow, Russia; Institute for Theoretical and Experimental Physics (ITEP), Moscow, Russia; National Research Nuclear University MEPhI, Moscow, Russia; D.V. Skobeltsyn Institute of Nuclear Physics, M.V. Lomonosov Moscow State University, Moscow, Russia; Fakultät für Physik, Ludwig-Maximilians-Universität München, Munich, Germany; Max-Planck-Institut für Physik (Werner-Heisenberg-Institut), Munich, Germany; Nagasaki Institute of Applied Science, Nagasaki, Japan; Graduate School of Science and Kobayashi-Maskawa Institute, Nagoya University, Nagoya, Japan; INFN Sezione di Napoli, Naples, Italy; Department of Physics and Astronomy, University of New Mexico, Albuquerque, NM USA; Institute for Mathematics, Astrophysics and Particle Physics, Radboud University Nijmegen/Nikhef, Nijmegen, The Netherlands; Nikhef National Institute for Subatomic Physics and University of Amsterdam, Amsterdam, The Netherlands; Department of Physics, Northern Illinois University, De Kalb, IL USA; Budker Institute of Nuclear Physics, SB RAS, Novosibirsk, Russia; Department of Physics, New York University, New York, NY USA; Ohio State University, Columbus, OH USA; Faculty of Science, Okayama University, Okayama, Japan; Homer L. Dodge Department of Physics and Astronomy, University of Oklahoma, Norman, OK USA; Department of Physics, Oklahoma State University, Stillwater, OK USA; Palacký University, RCPTM, Olomouc, Czech Republic; Center for High Energy Physics, University of Oregon, Eugene, OR USA; LAL, Université Paris-Sud and CNRS/IN2P3, Orsay, France; Graduate School of Science, Osaka University, Osaka, Japan; Department of Physics, University of Oslo, Oslo, Norway; Department of Physics, Oxford University, Oxford, UK; INFN Sezione di Pavia, Pavia, Italy; Department of Physics, University of Pennsylvania, Philadelphia, PA USA; Petersburg Nuclear Physics Institute, Gatchina, Russia; INFN Sezione di Pisa, Pisa, Italy; Department of Physics and Astronomy, University of Pittsburgh, Pittsburgh, PA USA; Laboratorio de Instrumentacao e Fisica Experimental de Particulas, LIP, Lisbon, Portugal; Institute of Physics, Academy of Sciences of the Czech Republic, Prague, Czech Republic; Czech Technical University in Prague, Prague, Czech Republic; Faculty of Mathematics and Physics, Charles University in Prague, Prague, Czech Republic; State Research Center Institute for High Energy Physics, Protvino, Russia; Particle Physics Department, Rutherford Appleton Laboratory, Didcot, UK; INFN Sezione di Roma, Rome, Italy; INFN Sezione di Roma Tor Vergata, Rome, Italy; INFN Sezione di Roma Tre, Rome, Italy; Faculté des Sciences Ain Chock, Réseau Universitaire de Physique des Hautes Energies-Université Hassan II, Casablanca, Morocco; DSM/IRFU (Institut de Recherches sur les Lois Fondamentales de l’Univers), CEA Saclay (Commissariat à l’Energie Atomique et aux Energies Alternatives), Gif-sur-Yvette, France; Santa Cruz Institute for Particle Physics, University of California Santa Cruz, Santa Cruz, CA USA; Department of Physics, University of Washington, Seattle, WA USA; Department of Physics and Astronomy, University of Sheffield, Sheffield, UK; Department of Physics, Shinshu University, Nagano, Japan; Fachbereich Physik, Universität Siegen, Siegen, Germany; Department of Physics, Simon Fraser University, Burnaby, BC Canada; SLAC National Accelerator Laboratory, Stanford, CA USA; Faculty of Mathematics, Physics and Informatics, Comenius University, Bratislava, Slovak Republic; Department of Physics, University of Cape Town, Cape Town, South Africa; Department of Physics, Stockholm University, Stockholm, Sweden; Physics Department, Royal Institute of Technology, Stockholm, Sweden; Departments of Physics and Astronomy and Chemistry, Stony Brook University, Stony Brook, NY USA; Department of Physics and Astronomy, University of Sussex, Brighton, UK; School of Physics, University of Sydney, Sydney, Australia; Institute of Physics, Academia Sinica, Taipei, Taiwan; Department of Physics, Technion: Israel Institute of Technology, Haifa, Israel; Raymond and Beverly Sackler School of Physics and Astronomy, Tel Aviv University, Tel Aviv, Israel; Department of Physics, Aristotle University of Thessaloniki, Thessaloníki, Greece; International Center for Elementary Particle Physics and Department of Physics, The University of Tokyo, Tokyo, Japan; Graduate School of Science and Technology, Tokyo Metropolitan University, Tokyo, Japan; Department of Physics, Tokyo Institute of Technology, Tokyo, Japan; Department of Physics, University of Toronto, Toronto, ON Canada; TRIUMF, Vancouver, BC Canada; Faculty of Pure and Applied Sciences, University of Tsukuba, Tsukuba, Japan; Department of Physics and Astronomy, Tufts University, Medford, MA USA; Centro de Investigaciones, Universidad Antonio Narino, Bogotá, Colombia; Department of Physics and Astronomy, University of California Irvine, Irvine, CA USA; INFN Gruppo Collegato di Udine, Sezione di Trieste, Udine, Italy; Department of Physics, University of Illinois, Urbana, IL USA; Department of Physics and Astronomy, University of Uppsala, Uppsala, Sweden; Instituto de Física Corpuscular (IFIC) and Departamento de Física Atómica, Molecular y Nuclear and Departamento de Ingeniería Electrónica and Instituto de Microelectrónica de Barcelona (IMB-CNM), University of Valencia and CSIC, Valencia, Spain; Department of Physics, University of British Columbia, Vancouver, BC Canada; Department of Physics and Astronomy, University of Victoria, Victoria, BC Canada; Department of Physics, University of Warwick, Coventry, UK; Waseda University, Tokyo, Japan; Department of Particle Physics, The Weizmann Institute of Science, Rehovot, Israel; Department of Physics, University of Wisconsin, Madison, WI USA; Fakultät für Physik und Astronomie, Julius-Maximilians-Universität, Würzburg, Germany; Fachbereich C Physik, Bergische Universität Wuppertal, Wuppertal, Germany; Department of Physics, Yale University, New Haven, CT USA; Yerevan Physics Institute, Yerevan, Armenia; Centre de Calcul de l’Institut National de Physique Nucléaire et de Physique des Particules (IN2P3), Villeurbanne, France; CERN, Geneva, Switzerland; Istanbul Aydin University, Istanbul, Turkey; Division of Physics, TOBB University of Economics and Technology, Istanbul, Turkey; Department of Physics, Dogus University, Istanbul, Turkey; Department of Physics Engineering, Gaziantep University, Gaziantep, Turkey; Dipartimento di Fisica e Astronomia, Università di Bologna, Bologna, Italy; Electrical Circuits Department, Federal University of Juiz de Fora (UFJF), Juiz de Fora, Brazil; Federal University of Sao Joao del Rei (UFSJ), Sao Joao del Rei, Brazil; Instituto de Fisica, Universidade de Sao Paulo, São Paulo, Brazil; Physics Department, National Institute for Research and Development of Isotopic and Molecular Technologies, Cluj Napoca, Romania; University Politehnica Bucharest, Bucharest, Romania; West University in Timisoara, Timisoara, Romania; Departamento de Física, Universidad Técnica Federico Santa María, Valparaiso, Chile; Department of Modern Physics, University of Science and Technology of China, Anhui, China; Department of Physics, Nanjing University, Jiangsu, China; School of Physics, Shandong University, Shandong, China; Department of Physics and Astronomy, Shanghai Key Laboratory for Particle Physics and Cosmology, Shanghai Jiao Tong University, Shanghai, China; Physics Department, Tsinghua University, Beijing, 100084 China; Dipartimento di Fisica, Università della Calabria, Rende, Italy; Marian Smoluchowski Institute of Physics, Jagiellonian University, Kraków, Poland; Dipartimento di Fisica, Università di Genova, Genova, Italy; High Energy Physics Institute, Tbilisi State University, Tbilisi, Georgia; Physikalisches Institut, Ruprecht-Karls-Universität Heidelberg, Heidelberg, Germany; ZITI Institut für technische Informatik, Ruprecht-Karls-Universität Heidelberg, Mannheim, Germany; Department of Physics, The University of Hong Kong, Pok Fu Lam, China; Department of Physics, The Hong Kong University of Science and Technology, Clear Water Bay, Kowloon, Hong Kong, China; Dipartimento di Matematica e Fisica, Università del Salento, Lecce, Italy; Dipartimento di Fisica, Università di Milano, Milan, Italy; Dipartimento di Fisica, Università di Napoli, Naples, Italy; Dipartimento di Fisica, Università di Pavia, Pavia, Italy; Dipartimento di Fisica E. Fermi, Università di Pisa, Pisa, Italy; Faculdade de Ciências, Universidade de Lisboa, Lisbon, Portugal; Department of Physics, University of Coimbra, Coimbra, Portugal; Centro de Física Nuclear da Universidade de Lisboa, Lisbon, Portugal; Departamento de Fisica, Universidade do Minho, Braga, Portugal; Departamento de Fisica Teorica y del Cosmos and CAFPE, Universidad de Granada, Granada, Portugal; Dep Fisica and CEFITEC of Faculdade de Ciencias e Tecnologia, Universidade do Minho, Braga, Portugal; Dipartimento di Fisica, Sapienza Università di Roma, Rome, Italy; Dipartimento di Fisica, Università di Roma Tor Vergata, Rome, Italy; Dipartimento di Matematica e Fisica, Università Roma Tre, Rome, Italy; Centre National de l’Energie des Sciences Techniques Nucleaires, Rabat, Morocco; Faculté des Sciences Semlalia, Université Cadi Ayyad, LPHEA-Marrakech, Marrakech, Morocco; Faculté des Sciences, Université Mohamed Premier and LPTPM, Oujda, Morocco; Faculté des Sciences, Université Cadi Ayyad, LPHEA-Marrakech, Marrakech, Morocco; Department of Subnuclear Physics, Institute of Experimental Physics of the Slovak Academy of Sciences, Kosice, Slovak Republic; Department of Physics, University of Johannesburg, Johannesburg, South Africa; School of Physics, University of the Witwatersrand, Johannesburg, South Africa; The Oskar Klein Centre, Stockholm, Sweden; Department of Physics and Astronomy, York University, Toronto, ON Canada; ICTP, Trieste, Italy; Fisica e Ambiente, Università di Udine, Udine, Italy

## Abstract

A search for a new resonance decaying to a *W* or *Z* boson and a Higgs boson in the $\ell \ell / \ell \nu / \nu \nu + b \bar{b}$ final states is performed using 20.3 fb$^{-1}$ of *pp* collision data recorded at $\sqrt{s}=$ 8 TeV with the ATLAS detector at the Large Hadron Collider. The search is conducted by examining the *WH* / *ZH* invariant mass distribution for a localized excess. No significant deviation from the Standard Model background prediction is observed. The results are interpreted in terms of constraints on the Minimal Walking Technicolor model and on a simplified approach based on a phenomenological Lagrangian of Heavy Vector Triplets.

## Introduction

Although the Higgs boson discovery by the ATLAS [1] and CMS [2] collaborations imposes strong constraints on theories beyond the Standard Model (SM), the extreme fine tuning in quantum corrections required to have a light fundamental Higgs boson [3, 4] suggests that the SM may be incomplete, and not valid beyond a scale of a few TeV. Various dynamical electroweak symmetry breaking scenarios which attempt to solve this naturalness problem, such as Minimal Walking Technicolor [5–8], Little Higgs [9], or composite Higgs models [10, 11], predict the existence of new resonances decaying to a vector boson plus a Higgs boson.

Using the full dataset collected by the ATLAS detector at 8 TeV centre-of-mass energy at the Large Hadron Collider, a search is performed for a heavy resonance decaying to *VH*, where *V* is a *W* or *Z* boson and *H* is the SM Higgs boson. This analysis looks for the leptonic decay of the *W* or *Z* boson and the Higgs decay into a *b*-quark pair. Therefore the selected final states are: zero charged leptons targeting $Z(\rightarrow \nu \nu )b{\bar{b}}$ decays, one charged lepton $W(\rightarrow \ell \nu )b{\bar{b}}$, and two oppositely charged leptons $Z(\rightarrow \ell \ell )b{\bar{b}}$ where $\ell =e, \mu$. The search is performed by examining the distribution of the reconstructed *VH* mass ($m_{VH}$) for a localized excess. The signal strength and the background normalization are determined from a likelihood fit to the data distribution in the three channels studied.

As a benchmark, the Minimal Walking Technicolor model (MWT) is used, a model with strongly coupled dynamics. This model predicts two triplets of resonances, $R_1^{\pm ,0}$ and $R_2^{\pm ,0}$, one of which is a vector and the other an axial-vector, that couple to vector bosons with strength $\tilde{g}$ and to fermions with $g/\tilde{g}$, where *g* is the weak SU(2) coupling constant. The bare axial-vector mass $m_A$ determines the masses of $R_1$ and $R_2$, with the lower mass resonance $R_1$ having a mass close to $m_A$. Recent lattice simulations in this model [12–14] predict masses close to 2 TeV. The decay channels $R_{1,2}^{\pm } \rightarrow WH$ and $R_{1,2}^{0} \rightarrow ZH$, lead to $Wb{\bar{b}}$ and $Zb{\bar{b}}$ final states.

A simplified approach based on a phenomenological Lagrangian [15] that incorporates Heavy Vector Triplets (HVT), which allows the interpretation of the results in a model-independent way, is also used. Here, the new heavy vector bosons, $V'^{\pm ,0}$, couple to the Higgs and SM gauge bosons via a combination of parameters $g_Vc_H$ and to the fermions via the combination $(g^2/g_V)~c_F$. The parameter $g_V$ represents the strength of the new vector boson interaction, while $c_H$ and $c_F$, which represent the couplings to the Higgs and the fermions respectively, are expected to be of order unity in most models. Two benchmark models [15] are used here. In the first model, referred to as *model A*, the branching fractions to fermions and gauge bosons are comparable, as in some extensions of the SM gauge group [16]. For *model B*, fermionic couplings are suppressed, as for example in a composite Higgs model [17].

The three final states presented in this Letter have been extensively studied for non-resonant production in ATLAS [18]. Moreover, a search for a pseudoscalar resonance in the $\ell \ell b {\bar{b}}$ and $\nu \nu b {\bar{b}}$ channels has already been published by ATLAS, setting limits on two-Higgs-doublet models [19]. Other searches for particles occurring in MWT and HVT models have been conducted by the ATLAS [20, 21] and CMS [22] collaborations.

## The ATLAS detector

The ATLAS detector [23] is a general-purpose particle detector used to investigate a broad range of physics processes. It includes inner tracking devices surrounded by a superconducting solenoid, electromagnetic and hadronic calorimeters and a muon spectrometer. The inner detector (ID) provides precision tracking of charged particles with pseudorapidity^1^  $|\eta | < 2.5$. The calorimeter system covers the pseudorapidity range $|\eta | < 4.9$. It is composed of sampling calorimeters with either liquid argon (LAr) or scintillator tiles as the active medium. The muon spectrometer consists of three large superconducting toroids and a system of trigger chambers and precision tracking chambers that provide triggering and tracking capabilities in the ranges of $|\eta | <$ 2.4 and $|\eta | <$2.7 respectively.

The ATLAS detector has a three-level trigger system to select events for offline analysis.

## Data and Monte Carlo samples

This analysis is based on $\sqrt{s}=8 {\mathrm{ TeV}}$*pp* collision data corresponding to 20.3 $\pm$ 0.6 fb$^{-1}$  [24]. The data used in the $\ell \nu b{\bar{b}}$ final state were collected using single-electron and single-muon triggers with transverse momentum ($p_{\mathrm{T}}\,$) thresholds from 24 to 60 GeV. The data used in the $\ell \ell b{\bar{b}}$ final state were collected using a combination of single-electron, single-muon, dielectron (*ee*) and dimuon ($\mu \mu$) triggers. The $p_{\mathrm{T}}\,$ thresholds for the *ee* and $\mu \mu$ triggers vary from 12 to 13 GeV. The data used in the $\nu \nu b{\bar{b}}$ final state were collected using a trigger that requires a missing transverse momentum ($\mathbf {E_{\mathrm{T}}^{\mathrm{miss}}\,}$) with magnitude $E_{T}^{\mathrm{miss}}$ greater than 80 GeV.

Simulated Monte Carlo (MC) samples for the MWT benchmark model use the implementation [25] in Madgraph5 [26], with the Higgs boson mass set to 126 GeV. The parameter $\tilde{g}$ is set to 2 for signal generation. Constraints on other values of this parameter can be set using the same samples since the kinematic distributions do not depend on $\tilde{g}$. The parameter *S*, which is an approximate value [27] of the Peskin–Takeuchi *S* parameter [28] which measures potential new contributions to electroweak radiative corrections, is set to 0.3, in accordance with the recommendations in Ref. [29].

Signal samples for the HVT model are also generated with Madgraph5. The parameter $c_F$ is assumed to be the same for quarks and leptons including third-generation fermions. Other parameters involving more than one heavy vector boson, $g_Vc_{VVV}$, $g_V^2 c_{VVHH}$ and $c_{VVW}$, have negligible effect on the overall cross sections for the processes of interest here. For all signal events, parton showering and hadronization is performed with Pythia8 [30, 31] and the CTEQ6L1 [32] parton distribution functions (PDFs) are used. Benchmark signal samples are generated for a range of resonance masses from 300 to $2000\,\hbox {GeV}$ in steps of 100 GeV.

MC samples are used to model the shape and normalization of most SM background processes, although some are later adjusted using data-based corrections extracted from control samples. The production of *W* and *Z* bosons in association with jets is simulated with Sherpa 1.4.1 [33] using the CT10 PDFs [34]. Top quark pair production is simulated using Powheg [35, 36] with the Powheg-BOX program [37] interfaced to Pythia6, using the CTEQ6L1 PDFs. In this analysis, the final normalizations of these dominant backgrounds are constrained by the data, but theoretical cross sections are used to optimize the selection. The cross sections are calculated at NNLO accuracy for *W* / *Z*+jets [38] and at NNLO+NNLL accuracy for $t\bar{t}$ [39]. Single top quark production is simulated with Powheg and AcerMC [40] interfaced to Pythia6, using the CTEQ6L1 PDFs, and the cross sections are taken from Ref. [41]. Diboson production (*WW*,*WZ*,*ZZ*) is simulated using Powheg interfaced to Pythia8, using the CT10 PDFs, and the cross sections are obtained at NLO from mcfm [42]. Finally, SM Higgs boson production in association with a *W* / *Z* boson is simulated using Pythia8 with the CTEQ6L1 PDFs, and considered as a background in this search. It is scaled to the SM cross section [18].

All MC simulated samples include the effect of multiple *pp* interactions in the same and neighbouring bunch crossings (pile-up) by overlaying simulated minimum-bias events on each generated signal or background event. The number of overlaid events is such that the distribution of the number of interactions per *pp* bunch crossing in the simulation matches that observed in the data, with on average 21 interactions per bunch crossing. The generated samples are processed through the Geant4-based ATLAS detector simulation [43, 44] or a fast simulation using a parameterization of the performance of the calorimetry and Geant4 for the other parts of the detector [45]. Simulated events are reconstructed with the standard ATLAS reconstruction software used for collision data.

## Object reconstruction

The physics objects used in this analysis are electrons, muons, jets and missing transverse momentum.

Electrons are identified for $|\eta | < 2.47$ and $p_{\mathrm{T}}\,> 7$ GeV from energy clusters in the electromagnetic calorimeter that are matched to tracks in the inner detector [46]. Quality requirements based on the calorimeter cluster and track are applied to reduce contamination from jets.

Muons are reconstructed in the muon spectrometer in the range $|\eta |< 2.7$ and $p_{\mathrm{T}}\,> 4$ GeV [47]. For $|\eta |< 2.5$ the muon spectrometer track must be matched with a track in the inner detector and information from both is used to reconstruct the momentum. Muons considered for this analysis must have $p_{\mathrm{T}}\,> 7$ GeV.

Lepton candidates are required to be isolated to reduce the multijet background. The scalar sum of the transverse momenta of tracks with $p_{\mathrm{T}}\,>1\,\hbox {GeV}$ within a cone of $\Delta R=\sqrt{(\Delta \eta )^2+(\Delta \phi )^2}=0.2$ around the lepton track (tracking isolation) is required to be less than 10 % of the lepton $p_{\mathrm{T}}\,$.

Jets are reconstructed using the anti-$k_t$ algorithm [48] with radius parameter $R = 0.4$. The jet transverse momentum is corrected for energy losses in passive material, for the non-compensating response of the calorimeter, and for any additional energy due to multiple *pp* interactions [49]. Jets are required to have $p_{\mathrm{T}}\,>30\,\hbox {GeV}$ and $|\eta |<4.5$. To reject low-$p_{\mathrm{T}}\,$ jets from pile-up, for jets with $p_{\mathrm{T}}\,<50\,\hbox {GeV}$ and $|\eta |<2.5$, the scalar sum of the $p_{\mathrm{T}}\,$ of associated tracks, originating from the reconstructed primary vertex, is required to be at least 50 % of the scalar sum of the $p_{\mathrm{T}}\,$ of all associated tracks. To avoid double-counting of leptons and jets, an overlap removal procedure is applied [18].

In the pseudorapidity range $|\eta |<2.5$, jets originating from *b*-quarks are identified using a multi-variate *b*-tagging algorithm [50]. This has an efficiency of 70 % and a misidentification rate of less than 1 % for selecting jets initiated by light quarks or gluons and of about 20 % for jets initiated by *c*-quarks, as determined from $t{\bar{t}}$ MC events.

The missing transverse momentum is calculated as the negative of the vectorial sum of the calorimeter-based transverse momenta of all electrons, jets, and calibrated calorimeter clusters within $|\eta | < 4.9$ that are not associated with any other objects [51], as well as muon momenta. In addition, a track-based missing transverse momentum ($\mathbf {p_{{\text {T}}}^{{{\text {miss}}}}}$, with magnitude $p_{{\text {T}}}^{{{\text {miss}}}}$ ) is used, calculated as the negative vectorial sum of the track-based transverse momenta of objects with $|\eta | < 2.4$ associated with the primary vertex.

## Event selection and reconstruction

Events are categorized into the ${\nu }{\nu } b{\bar{b}}, {\ell }{\nu } b{\bar{b}} \hbox {or} {\ell }{\ell } b{\bar{b}}$ channels if they have zero, one or two reconstructed charged leptons respectively. All categories require at least two jets in the pseudorapidity range $|\eta |< 2.5$ (central jets). The channels are further subdivided into categories of events containing one or two *b*-tagged jets; events with zero or $\ge 3$*b*-tagged jets are rejected. The Higgs boson candidate (and its mass $m_{b{\bar{b}}}$) is reconstructed from the two *b*-tagged jets or, for 1-*b*-tag events, the *b*-tagged jet and the highest-$p_{\mathrm{T}}\,$ remaining central jet. In order to suppress *W* / *Z*+jets background, at least one of the jets must have $p_{\mathrm{T}}\,>45$ GeV and the invariant mass of the dijet pair must be in the range $105<m_{b{\bar{b}}}<145$ GeV, consistent with the Higgs mass. In order to reduce the $t\bar{t}$ background in the $\nu \nu b{\bar{b}}$ and $\ell \nu b{\bar{b}}$ channels, events are rejected if they contain four or more jets. To improve the resolution of the *VH* mass a constraint to the Higgs boson mass is applied by scaling the Higgs boson candidate jet momenta by $m_{H}/m_{b{\bar{b}}}$ ($m_{H}=125\,\text {GeV}$). Further channel-specific cuts are applied as outlined below.

### $\nu \nu b{\bar{b}}$ channel

Events are selected with $E_{\mathrm{T}}^{\mathrm{miss}}\,>120$ GeV and $p_{{\text {T}}}^{{{\text {miss}}}}>30\,$GeV. A requirement is made on $H_{\mathrm{T}}$, defined as the scalar sum of the $p_{\mathrm{T}}\,$ of all jets, in order to keep a high trigger efficiency: $H_{\mathrm{T}}>120\,$GeV (>$150\,$ GeV) for events with two (three) jets. Selections are also applied on the angle between the jets used for reconstructing the Higgs candidate, $\Delta R_{b{\bar{b}}}$, to suppress the $W/Z+$jets background [18]: for $120 < E_{\mathrm{T}}^{\mathrm{miss}}\,< 160\,$GeV, $0.7< \Delta R_{b{\bar{b}}} <1.8$; for $160 < E_{\mathrm{T}}^{\mathrm{miss}}\,< 200\,$GeV, $\Delta R_{b{\bar{b}}}< 1.8$; for $E_{\mathrm{T}}^{\mathrm{miss}}\,>200\,$GeV, $\Delta R_{b{\bar{b}}}<1.4$. Events containing an electron or muon passing the selection cuts described in Sect. 4 are removed.

In events with real $E_{\mathrm{T}}^{\mathrm{miss}}\,\,$ the directions of $\mathbf{E}_{{\mathbf{T}}}^{\mathbf{miss}}$ and $\mathbf {p_{{\text {T}}}^{{{\text {miss}}}}}$ are expected to be similar. In events with fake $E_{\mathrm{T}}^{\mathrm{miss}}\,$ arising from a jet energy fluctuation, the direction of $\mathbf {E_{\mathrm{T}}^{\mathrm{miss}}\,}$ should be close to the direction of the poorly measured jet. Therefore additional criteria are imposed on angular quantities in order to suppress the multijet background: the azimuthal angle between $\mathbf {E_{\mathrm{T}}^{\mathrm{miss}}\,}$ and $\mathbf {p_{{\text {T}}}^{{{\text {miss}}}}}$, $\Delta \phi (\mathbf {E_{\mathrm{T}}^{\mathrm{miss}}\,}, \mathbf {p_{{\text {T}}}^{{{\text {miss}}}}})<\pi /2$; the minimum azimuthal angle between $\mathbf {E_{\mathrm{T}}^{\mathrm{miss}}\,}$ and any jet, $\mathrm{min}[\Delta \phi (\mathbf {E_{\mathrm{T}}^{\mathrm{miss}}\,},\mathrm{jet})] > 1.5$; and the azimuthal angle between $\mathbf {E_{\mathrm{T}}^{\mathrm{miss}}\,}$ and the jet pair combination used to reconstruct the Higgs candidate, $\Delta \phi (\mathbf {E_{\mathrm{T}}^{\mathrm{miss}}\,},b{\bar{b}})>2.8$.

It is not possible to accurately reconstruct the invariant mass of the *ZH* system due to the missing neutrinos, so the transverse mass is used as the final discriminant: $m^{{\mathrm{T}}}_{VH}= \sqrt{(E_{T}^{b{\bar{b}}} + {E_{\mathrm{T}}^{\mathrm{miss}}\,})^2 - ({\mathbf {p_{\mathrm{T}}\,^{b{\bar{b}}}}}+ \mathbf {E_{\mathrm{T}}^{\mathrm{miss}}\,})^2}$, where ${{{p_{\mathrm{T}}\,^{b{\bar{b}}}}}}$ is the transverse momentum of the Higgs candidate. The total acceptance times selection efficiency varies from 15 % for $m_{R_1} = 400$ GeV, to 30 % for $m_{R_1} = 1000$ GeV and down to 2 % for $m_{R_1} = 2000$ GeV. The drop at very high masses is due to the merging of the jets.

### $\ell \nu b{\bar{b}}$ channel

In order to suppress the multijet background and ensure the single-lepton triggers are fully efficient, tighter identification criteria are placed on the lepton in this channel. The lepton $p_{\mathrm{T}}\,$ requirement is raised to $p_{\mathrm{T}}\,>25$ GeV and, for the muon channel, the pseudorapidity is restricted to $|\eta |<2.5$. Moreover, the tracking isolation is tightened and required to be less than 4 % of the lepton $p_{\mathrm{T}}\,$. Similarly, the sum of transverse energy deposits in the calorimeter within a cone of $\Delta R=0.3$ around the lepton, excluding the transverse energy due to the lepton and the correction for the expected pile-up contribution, is required to be less than 4 % of the lepton $p_{\mathrm{T}}\,$.

The multijet background is further reduced by requiring $\Delta \phi (\mathbf {E_{\mathrm{T}}^{\mathrm{miss}}\,},\mathrm{jet})>1.0$. *W* boson candidates are selected by requiring $E_{\mathrm{T}}^{\mathrm{miss}}\,>30$ GeV and the transverse mass reconstructed from the lepton and $E_{\mathrm{T}}^{\mathrm{miss}}\,$, $m^\mathrm{T}_W = \sqrt{2 \times E_\mathrm{T}^{\ell } \times E_{\mathrm{T}}^{\mathrm{miss}}\,\times (1-\text {cos}\Delta \phi (\ell , \mathbf {E_{\mathrm{T}}^{\mathrm{miss}}\,})}) >20$ GeV.

The *WH* system mass, $m_{VH}$, is reconstructed from the lepton, the $E_{\mathrm{T}}^{\mathrm{miss}}\,$ and the two jets. The momentum of the neutrino in the *z*-direction, $p_{z}$, is obtained by imposing the *W* boson mass constraint on the lepton and neutrino system, which leads to a quadratic equation. Here $p_z$ is taken as either the real component of the complex solutions or the smaller of the two real solutions.

In order to reduce the $W+$jets background, a requirement is imposed on the transverse momentum of the *W* boson, $p_{\text {T}}^{W}>0.4 \times m_{VH}$. The cut depends on $m_{VH}$ since the background is generally produced at low $p_{\text {T}}^{W}$, whereas for signal the mean $p_{\text {T}}^W$ increases with $m_{VH}$. The total acceptance times selection efficiency varies from 8 % for $m_{R_1} = 400$ GeV, to 20 % for $m_{R_1} = 1000$ GeV and down to 2 % for $m_{R_1} = 2000$ GeV.

### $\ell \ell b{\bar{b}}$ channel

Events in this channel are selected by requiring two reconstructed leptons of the same flavour with opposite charge. In order to reduce the multijet background while keeping a high signal acceptance, tighter requirements are placed on one of the leptons. These tighter electrons or muons must have $p_{\mathrm{T}}\,>25$ GeV and, in addition, muons are restricted to $|\eta |<2.5$. A cut on the two-lepton invariant mass of 83 GeV$<m_{\ell \ell }<99$ GeV is imposed to reduce $t\bar{t}$ and multijet backgrounds. The $t\bar{t}$ background is further reduced by requiring $E_{\mathrm{T}}^{\mathrm{miss}}\,<60$ GeV.

The invariant mass of the two leptons and two jets is used to reconstruct $m_{VH}$.

In order to reduce the dominant $Z+$jets background, a selection, optimized for this channel, is imposed on the transverse momentum of the *Z* boson: $p_{\text {T}}^{Z}>0.4\times m_{VH}-100$ GeV. The total acceptance times selection efficiency varies from 18 % for $m_{R_1} = 400$ GeV, to 30 % for $m_{R_1} = 1000$ GeV and down to 1 % for $m_{R_1} = 2000$ GeV.

## Background estimation

All backgrounds except the multijet background are estimated from simulation, with data-based corrections for the dominant *W* / *Z*+jets background as described in the following. The rate and shape of the multijet (MJ) background are estimated with data-driven methods.

The MJ background is estimated in the 0-lepton channel using an “ABCD method” based on two uncorrelated variables: $\mathrm{min}[\Delta \phi (\mathbf {E_{\mathrm{T}}^{\mathrm{miss}}\,},\mathrm{jet})]$ and $\Delta \phi (\mathbf {E_{\mathrm{T}}^{\mathrm{miss}}\,}, \mathbf {p_{{\text {T}}}^{{{\text {miss}}}}})$. The data are divided into four regions such that three of the regions are dominated by background. The signal region (A) is defined as explained in Sect. 5. The MJ-dominated region C is obtained by reversing the $\Delta \phi (\mathbf {E_{\mathrm{T}}^{\mathrm{miss}}\,}, \mathbf {p_{{\text {T}}}^{{{\text {miss}}}}})$ requirement. An MJ template in region A is obtained using events in region C after subtracting the contribution of other backgrounds, taken from simulation. The template is then normalized by a fit to the regions with $\mathrm{min}[\Delta \phi (\mathbf {E_{\mathrm{T}}^{\mathrm{miss}}\,},\mathrm{jet})] < 0.4$ [18] (regions B and D with orthogonal $\Delta \phi (\mathbf {E_{\mathrm{T}}^{\mathrm{miss}}\,}, \mathbf {p_{{\text {T}}}^{{{\text {miss}}}}})$ requirements).

In the 1-lepton channel, the MJ background is determined separately for the electron and muon sub-channels. An MJ-background template is obtained from an MJ-dominated region after subtracting the small contribution from the other backgrounds. An MJ-dominated region is obtained by loosening the lepton identification requirements and reversing the isolation criteria. A binned fit of the full $E_{\mathrm{T}}^{\mathrm{miss}}\,$ spectrum of the data to the sum of the MJ contribution, *W* / *Z*+jets and other MC contributions is then used to extract the MJ normalization. The templates are validated in a control region enriched in MJ events, selected by reversing the $E_{\mathrm{T}}^{\mathrm{miss}}\,$ requirement.

For the 2-lepton channel in the $ee b {\bar{b}}$ final state, the MJ background shape is determined by selecting events with reversed electron isolation criteria and its normalization is extracted by fitting the full data $m_{ee}$ distribution including *Z* sidebands. The MJ background in the $\mu \mu b {\bar{b}}$ final state is found to be negligible.

The *W* / *Z*+jets simulated samples are split into different components according to the true flavour of the jets, i.e. $W/Z+qq$, $W/Z+cq$, where *q* denotes a light quark (*u*, *d*, *s*) or a gluon, and *W* / *Z* plus heavy flavour (hf). The latter includes: $W/Z+b{\bar{b}}$, $W/Z+bq$,$W/Z+bc$, $W/Z+cc$. The normalizations of $W+cq$, $Z+cq$ and $W+$hf, $Z+$hf are free parameters of the global likelihood fit. The scale factors after the fit are all consistent with 1, except for the Z+hf normalization that is 15 % higher as seen in previous measurements [18]. The *W* / *Z*+jets modelling is checked in control regions selected by requiring events with no *b*-tagged jets or in the $m_{b{\bar{b}}}$ sideband region in the 1-tag and 2-tag channels. A difference between data and simulation is observed in the 0-tag control region and a correction is extracted as a function of the azimuthal angle difference between the two leading-$p_{\mathrm{T}}\,$ jets, $\Delta \phi ({\text {jet}}_{1} ,{\text {jet}}_{2})$. This is used to reweight the $Z+qq$ and $W+qq$ components. After this correction is applied a discrepancy is observed in the $p_{\mathrm{T}}\,^{\ell \ell }$ distribution in the 2-lepton channel after the requirement of at least one *b*-tagged jet. A correction is extracted and used to reweight the $Z+cq$ and $Z+$hf components. The full procedure is described in detail in Ref. [18].

The background contributions from single top quark and diboson production are normalized to the number of background events predicted by simulation while the $t\bar{t}$ normalization is a free parameter in the likelihood fit. The description of the shape of the $t \bar{t}$ background from MC simulation has been validated in samples dominated by top pair events. Good agreement within uncertainties is observed between data and expectation in these validation regions.

The $t \bar{t}$ control region is defined by requiring exactly one electron and one muon, one of which has $p_{\mathrm{T}}\,>25$ GeV, and two *b*-tagged jets. It is included in the likelihood fit to constrain the $t \bar{t}$ normalization. The scale factor for the $t\bar{t}$ normalization is found to be $1.03 \pm 0.04$ after the likelihood fit to the 0- and 2-lepton channel plus the $t \bar{t}$ control region, and $0.99 \pm 0.09$ from the fit to the 1-lepton channel. The fit procedure is described in more detail in Sect. 8.

## Systematic uncertainties

The most important experimental systematic uncertainties come from the jet energy scale (JES) and *b*-tagging efficiency.

The JES systematic uncertainty arises from several sources including uncertainties from the in-situ calibration, the corrections dependent on pile-up and the jet flavour composition [52]. The fractional systematic uncertainty on the JES ranges from 3 % for a 20 GeV jet to 1 % for a 1 TeV jet.

The uncertainty due to the jet energy resolution is also considered. It varies from 20 % for a jet with $p_{\mathrm{T}}\,>$ 20 GeV to 5 % for a jet with $p_{\mathrm{T}}\,>$ 1 TeV. The jet energy scale and resolution uncertainties are propagated to the reconstructed $\mathbf {E_{\mathrm{T}}^{\mathrm{miss}}\,}$. The uncertainty on $\mathbf {E_{\mathrm{T}}^{\mathrm{miss}}\,}$ also has a contribution from hadronic energy that is not included in jets [53].

The *b*-tagging efficiency uncertainty depends on jet $p_{\mathrm{T}}\,$ and comes mainly from the uncertainty on the measurement of the efficiency in $t\bar{t}$ events [50]. Uncertainties are also derived for *c*- and light-flavour jet tagging [54].

Other experimental systematic uncertainties that have a smaller impact are those on the lepton energy scale and identification efficiency and the efficiency of the triggers.

In addition to the experimental systematic uncertainties, uncertainties are taken into account for possible differences between data and the simulation model that is used for each process. For the background modelling uncertainties the procedure described in Ref. [18] is followed. The *Z*+jets and *W*+jets backgrounds include uncertainties on the relative fraction of the different flavour components, and shape uncertainties on the modelling of $m_{b{\bar{b}}}$, $\Delta \phi ({\text {jet}}_{1} ,{\text {jet}}_{2})$ and $p_{\mathrm{T}}\,^{Z}$ distributions. For $t\bar{t}$ production, shape uncertainties are included for the modelling of top quark transverse momentum, $m_{b{\bar{b}}}$ and $m_{VH}$ distributions. The uncertainty on the MJ background shape in the 1-lepton channel is evaluated by using alternative templates obtained by changing the definition of the data sidebands. The uncertainty on the MJ background normalization is taken to be 100, 30 and 50 % for the 0-, 1- and 2-lepton channels, respectively. These are extracted from fits using alternative templates.

The dominant uncertainties on the signal acceptance arise from the choice of PDFs (2–5 %) estimated by comparing the default PDFs to other sets, and from the factorization and renormalization scales (5–10 %) obtained by varying these up and down by a factor of two.

**Fig. 1:**
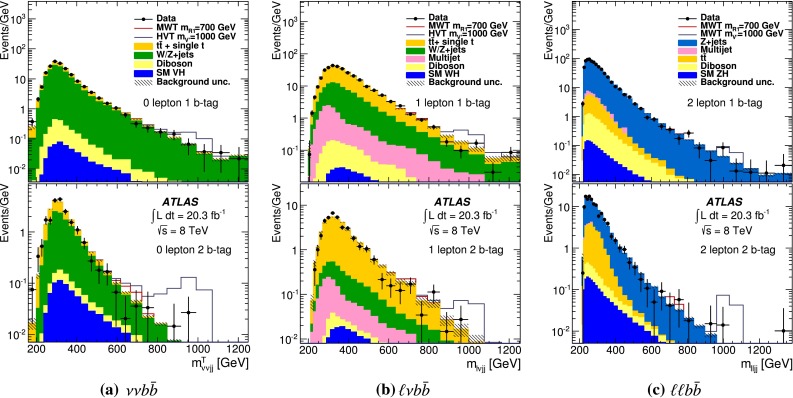
Distributions of the reconstructed, **a** transverse mass $m^{\text {T}}_{\nu \nu jj}$ for the $\nu \nu b {\bar{b}}$ final state, **b** invariant mass $m_{\ell \nu jj}$ for the $\ell \nu b {\bar{b}}$ final state and **c** invariant mass $m_{\ell \ell jj}$ for the $\ell \ell b {\bar{b}}$ final state for the 1-*b*-tag (*upper*) and 2-*b*-tag (*lower*) channels. The background expectation is shown after the profile likelihood fit to the data. Any overflow is included in the last bin. The signals are shown stacked on top of the background and correspond to the benchmark models MWT with $m_{R1} = 700$ GeV and HVT with $m_{V'} = 1000$ GeV normalized to the expected cross sections

**Fig. 2:**
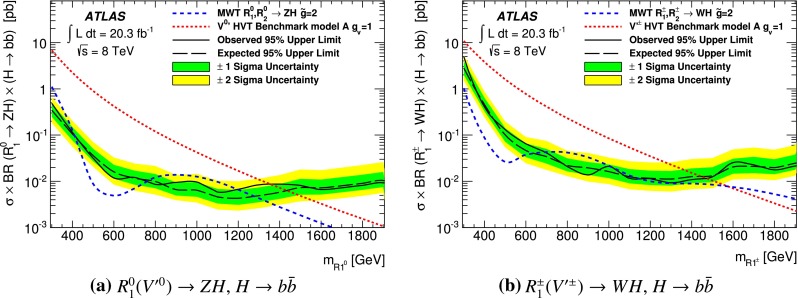
Combined upper limits at the 95 % CL for **a** the production cross section of $R_1^{0}$ ($V'^{0}$) times its branching ratio to *ZH* and branching ratio of *H* to $b{\bar{b}}$ and **b** the production cross section of $R_1^{\pm }$ ($V'^{\pm }$) times its branching ratio to *WH* and branching ratio of *H* to $b{\bar{b}}$ . The experimental limits are obtained using samples with a single resonance $R_1$; however, the theory curve line for MWT includes both $R_1$ and $R_2$. The dip near 500 GeV in this theory curve is due to the interference between $R_1$ and $R_2$ [7]

## Results and limit extraction

The reconstructed mass distributions for events passing the selection are shown in Fig. 1. The background expectation is shown after the profile likelihood fit to the data. Table 1 shows the number of events expected and observed in each final state.

**Table 1:**
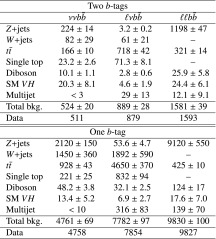
The number of expected and observed events for the three final states. The expectation is shown after the profile likelihood fit to the data. The quoted uncertainties are the combined systematic and statistical uncertainties. The overall background is more constrained than the individual components, causing the errors of individual components to be anti-correlated

No significant excess of events is observed in the data compared to the prediction from SM background sources. Exclusion limits at the 95 % confidence level (CL) are set on the production cross section times the branching fraction for MWT and HVT models. The limits for the charged resonance are obtained by performing the likelihood fit over the $\ell \nu b{\bar{b}}$ channel alone, while the $\ell \ell b{\bar{b}}$, $\nu \nu b{\bar{b}}$ channels as well as the $t\bar{t}$ control region are used for the neutral resonance.

**Fig. 3:**
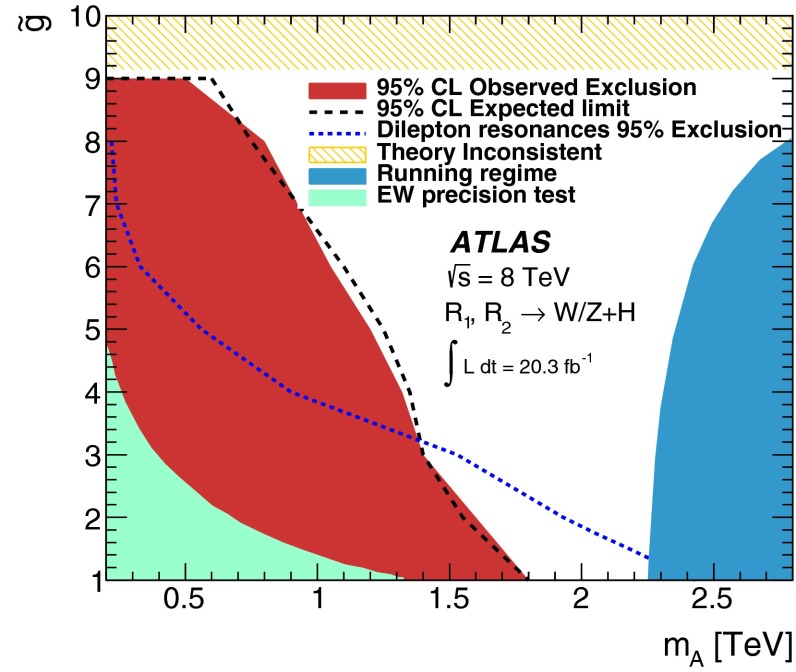
Exclusion contours at 95 % CL in the plane of the Minimal Walking Technicolor parameter space defined by the bare axial-vector mass versus the strength of the spin-1 resonance interaction {$m_A$, $\tilde{g}$}. Electroweak precision measurements exclude the (*green*) area in the *bottom left corner*. The requirement to stay in the walking regime excludes the (*blue*) area in the *right corner*. The *large* (*red*) *area* (*black dashed line*) shows the observed (expected) exclusion. The *blue dashed line* shows the observed exclusion from the dilepton resonance search [21]. The *upper region* is excluded due to non-real axial and axial-vector decay constants. Here both resonances predicted by MWT, $R_{1}$ and $R_{2}$, are fitted simultaneously

The exclusion limits are calculated with a modified frequentist method [55], also known as $CL_{s}$, and the profile-likelihood test statistic [56], using the binned $m_{\text {VH}}$ mass distributions for $\ell \nu b{\bar{b}}$, $\ell \ell b{\bar{b}}$ and $\nu \nu b{\bar{b}}$ final states. Systematic uncertainties and their correlations are taken into account as nuisance parameters. None of the systematic uncertainties considered are significantly constrained or pulled in the likelihood fit. Figure 2 shows 95 % CL upper limits on the production cross section multiplied by the branching fraction into *WH* and *ZH* as a function of the resonance mass separately for the charged $R_1^{\pm }$ and for the neutral $R_1^{0}$. The experimental limits are obtained using samples with a single resonance $R_1$, where the cross section for $R_2$ has been set to zero to be less model-dependent. The theoretical predictions for the HVT benchmark *model A* with coupling constant $g_{V}=1$ allow exclusion of $m_{V'^{0}} <1360$ GeV ($m_{V'^{\pm }} <1470$ GeV). For the MWT model, since there are two resonances of different mass, the results are displayed for the first one, $R_1^{0,\pm }$. The excluded regions are $m_{R_1^{0}}<410$ GeV, $750<m_{R_1^{0}}<1200$ GeV ($700<m_{R_1^{\pm }}<1150$ GeV). The dip near 500 GeV in this theory curve is due to the interference between $R_1$ and $R_2$ [7]. To study the scenario in which the masses of charged and neutral resonances are the same, a combined likelihood fit over all signal regions and the $t\bar{t}$ control region is also performed. The exclusion contours in the {$m_{A}$,$\tilde{g}$} plane for MWT are presented in Fig. 3. For this result, both resonances predicted by MWT, $R_1$ and $R_2$, are fitted simultaneously and, at each $\tilde{g}$, the different branching ratios to *WH* and *ZH* are taken into account. Electroweak precision data, a requirement to remain within the walking technicolor regime and constraints from requiring real-valued physical decay constants exclude a portion of the plane. This analysis is particularly sensitive at high $\tilde{g}$ values, where the limits exceed those from the dilepton resonance search [21].

**Fig. 4:**
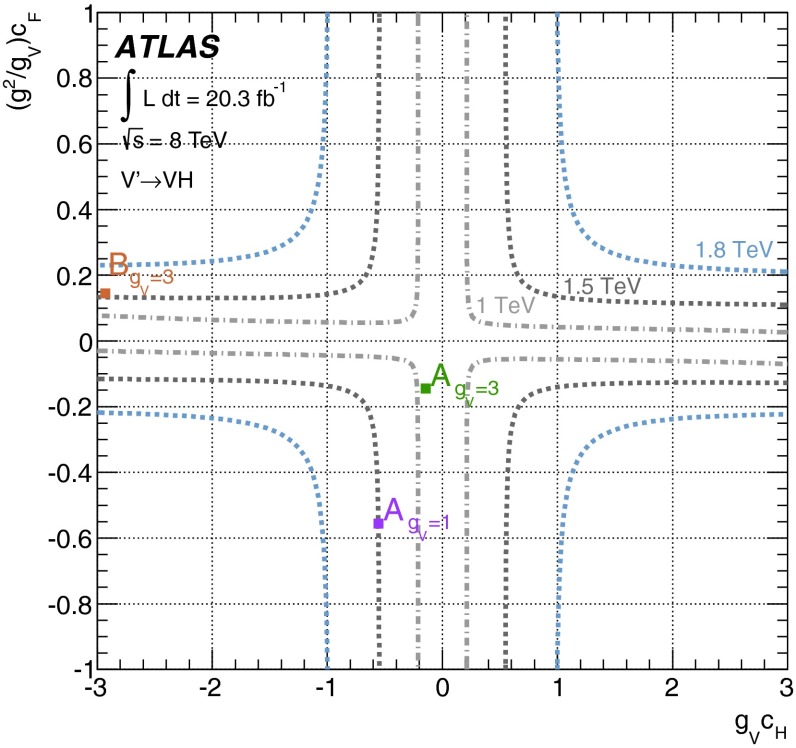
Observed 95 % CL exclusion contours in the HVT parameter space {($g^2$/$g_V$)$c_F$, $g_Vc_H$} for resonances of mass 1 ${\mathrm {TeV}}$, 1.5 ${\mathrm {TeV}}$ and 1.8 ${\mathrm {TeV}}$ . The areas outside the curves are excluded. Also shown are the benchmark model parameters A($g_V$=1), A($g_V$=3) and B($g_V$=3)

The exclusion contours in the HVT parameter space $\{(g^2/g_V)c_F, g_Vc_H\}$ for resonances of mass 1, 1.5 and 1.8 ${\mathrm {TeV}}$ are shown in Fig. 4 where all three channels are combined, taking into account the branching ratios to *WH* and *ZH* from the HVT model. These contours are produced by scanning the parameter space, using the HVT tools provided in a web-interface [15, 57].

## Summary

A search for a new heavy resonance decaying to *WH* / *ZH* is presented in this Letter. The search is performed using 20.3 fb$^{-1}$ of *pp* collision data at 8 TeV centre-of-mass energy collected by the ATLAS detector at the Large Hadron Collider. No significant deviations from the SM background predictions are observed in the three final states considered: $\ell \ell b {\bar{b}}$, $\ell \nu b {\bar{b}}$, $\nu \nu b {\bar{b}}$. Upper limits are set at the 95 % confidence level on the production cross sections of $R_1$ and $V'$ for the Minimal Walking Technicolor and Heavy Vector Triplets models respectively. Exclusion contours at 95 % CL in the MWT parameter space {$m_{A}$,$\tilde{g}$} and in the HVT parameter space {($g^2$/$g_V$)$c_F$, $g_Vc_H$} are presented.

## Footnotes

1 ATLAS uses a right-handed coordinate system with its origin at the nominal interaction point (IP) in the centre of the detector and the *z*-axis along the beam pipe. The *x*-axis points from the IP to the centre of the LHC ring, and the *y*-axis points upward. Cylindrical coordinates $(r,\phi )$ are used in the transverse plane, $\phi$ being the azimuthal angle around the beam pipe. The pseudorapidity is defined in terms of the polar angle $\theta$ as $\eta =-\ln \tan (\theta /2)$.

## References

[1] ATLAS Collaboration, Phys. Lett. B **716**, 1–29 (2012). arXiv:1207.7214 [hep-ex]

[2] CMS Collaboration, Phys. Lett. B **716**, 30–61 (2012). arXiv:1207.7235 [hep-ex]

[3] Particle Data Group Collaboration, K. Olive et al., Chin. Phys. C **38**, 090001 (2014)

[4] Wilson KG (1971). Phys. Rev. D.

[5] Sannino F, Tuominen K (2005). Phys. Rev. D.

[6] Foadi R, Frandsen MT, Ryttov TA, Sannino F (2007). Phys. Rev. D.

[7] Belyaev A (2009). Phys. Rev. D.

[8] Cacciapaglia G, Sannino F (2014). JHEP.

[9] M. Schmaltz, D. Tucker-Smith, Ann. Rev. Nucl. Part. Sci. **55**, 229–270 (2005). arXiv:hep-ph/0502182 [hep-ph]

[10] Dugan MJ, Georgi H, Kaplan DB (1985). Nucl. Phys. B.

[11] K. Agashe, R. Contino, A. Pomarol, Nucl. Phys. B **719**, 165–187 (2005). arXiv:hep-ph/0412089 [hep-ph]

[12] Fodor Z (2012). Phys. Lett. B.

[13] Z. Fodor, K. Holland, J. Kuti, D. Nogradi, C. H. Wong, PoS LATTICE **2013**, 062 (2014). arXiv:1401.2176 [hep-lat]

[14] Hietanen A, Lewis R, Pica C, Sannino F (2014). JHEP.

[15] D. Pappadopulo, A. Thamm, R. Torre, A. Wulzer, arXiv:1402.4431 [hep-ph]

[16] Barger VD, Keung W-Y, Ma E (1980). Phys. Rev. D.

[17] Contino R, Marzocca D, Pappadopulo D, Rattazzi R (2011). JHEP.

[18] ATLAS Collaboration, JHEP **1501**, 069 (2015). arXiv:1409.6212 [hep-ex]

[19] ATLAS Collaboration, arXiv:1502.04478 [hep-ex]

[20] ATLAS Collaboration, Phys. Lett. B **737**, 223–243 (2014). arXiv:1406.4456 [hep-ex]

[21] ATLAS Collaboration, Phys. Rev. D **90**(5), 052005 (2014). arXiv:1405.4123 [hep-ex]

[22] CMS Collaboration, arXiv:1502.04994 [hep-ex]

[23] ATLAS Collaboration, JINST **3**, S08003 (2008)

[24] ATLAS Collaboration, Eur. Phys. J. C **73**(8), 2518 (2013). arXiv:1302.4393 [hep-ex] 10.1140/epjc/s10052-013-2518-3 PMC4370906 25814867

[25] MWT Tools, http://cp3-origins.dk/research/units/tc-tools

[26] Alwall J, Herquet M, Maltoni F, Mattelaer O, Stelzer T (2011). JHEP.

[27] Foadi R, Sannino F (2013). Phys. Rev. D.

[28] Peskin ME, Takeuchi T (1992). Phys. Rev. D.

[29] Andersen JR, Hapola T, Sannino F (2012). Phys. Rev. D.

[30] T. Sjostrand, S. Mrenna, P. Z. Skands, JHEP **05**, 026 (2006). arXiv:hep-ph/0603175 [hep-ph]

[31] Sjostrand T, Mrenna S, Skands PZ (2008). Comput. Phys. Commun..

[32] Pumplin, J. et al., JHEP **0207**, 012 (2002). arXiv:hep-ph/0201195 [hep-ph]

[33] Gleisberg T (2009). JHEP.

[34] Lai H-L (2010). Phys. Rev. D.

[35] Nason P (2004). JHEP.

[36] Frixione S, Nason P, Oleari C (2007). JHEP.

[37] Alioli S, Nason P, Oleari C, Re E (2010). JHEP.

[38] Melnikov K, Petriello F (2006). Phys. Rev. D.

[39] Czakon M, Fiedler P, Mitov A (2013). Phys. Rev. Lett..

[40] B.P. Kersevan, E. Richter-Wa̧s, arXiv:hep-ph/0405247 [hep-ph]

[41] Kidonakis N (2011). Phys. Rev. D.

[42] Campbell JM, Ellis R (2010). Nucl. Phys. Proc. Suppl..

[43] GEANT4 Collaboration, S. Agostinelli et al., Nucl. Instrum. Meth. A **506**, 250–303 (2003)

[44] ATLAS Collaboration, Eur. Phys. J. C **70**, 823–874 (2010). arXiv:1005.4568 [physics.ins-det]

[45] ATLAS Collaboration, ATL-PHYS-PUB-2010-013 (2010). http://cdsweb.cern.ch/record/1300517

[46] ATLAS Collaboration, Eur. Phys. J. C **74**(7), 2941 (2014). arXiv:1404.2240 [hep-ex] 10.1140/epjc/s10052-014-2941-0 PMC4371047 25814900

[47] ATLAS Collaboration, Eur. Phys. J. C **74**(11), 3130 (2014). arXiv:1407.3935 [hep-ex] 10.1140/epjc/s10052-014-3130-x PMC4371046 25814875

[48] Cacciari M, Salam GP, Soyez G (2008). JHEP.

[49] ATLAS Collaboration, Eur. Phys. J. C **73**(3), 2304 (2013). arXiv:1112.6426 [hep-ex]

[50] ATLAS Collaboration, ATLAS-CONF-2014-004. http://cds.cern.ch/record/1664335

[51] ATLAS Collaboration, ATLAS-CONF-2010-057. http://cds.cern.ch/record/1281330

[52] ATLAS Collaboration, Eur. Phys. J. C **75**(1), 17 (2015). arXiv:1406.0076 [hep-ex]

[53] ATLAS Collaboration, ATLAS-CONF-2013-082. http://cds.cern.ch/record/1570993

[54] ATLAS Collaboration, ATLAS-CONF-2014-046. http://cds.cern.ch/record/1741020

[55] Read AL (2002). J. Phys. G.

[56] Cowan G, Cranmer K, Gross E, Vitells O (2011). Eur. Phys. J. C.

[57] HVT Tools, http://heidi.pd.infn.it/html/vector/

